# Influence of Modified Epoxy Resins on Peroxide Curing, Mechanical Properties and Adhesion of SBR, NBR and XNBR to Silver Wires. Part I: Application of Monoperoxy Derivative of Epoxy Resin (PO)

**DOI:** 10.3390/ma14051320

**Published:** 2021-03-09

**Authors:** Joanna Chudzik, Dariusz M. Bieliński, Michael Bratychak, Yuriy Demchuk, Olena Astakhova, Marcin Jędrzejczyk, Grzegorz Celichowski

**Affiliations:** 1 Institute of Polymer & Dye Technology, Lodz University of Technology, 90-924 Lodz, Poland; joanna.chudzik@edu.p.lodz.pl; 2 Institute of Chemistry and Chemical Technologies, Lviv Polytechnic National University, 79-013 Lviv, Ukraine; mbratychak@gmail.com (M.B.); yuriy_demchuk@ukr.net (Y.D.); KhTNH.dept@lpnu.ua (O.A.); 3 Institute of General and Ecological Chemistry, Lodz University of Technology, 90-924 Lodz, Poland; marcin.jedrzejczyk@p.lodz.pl; 4 Department of Materials Technology and Chemistry, Faculty of Chemistry, University of Lodz, 90-936 Lodz, Poland; grzegorz.celichowski@chemia.uni.lodz.pl

**Keywords:** rubber, epoxy resins, rubber modification, crosslinking, adhesion

## Abstract

The research was aimed at checking the effect of monoperoxy derivative of epoxy resin (PO) on the possibility of rubber crosslinking and a subsequent adhesion of the modified rubber to silver wires. Three of the commonly industrially used rubbers were selected for the study: styrene-butadiene rubber (SBR), acrylonitrile-butadiene rubber (NBR) and carboxylated acrylonitrile-butadiene rubber (XNBR), together with the popular, commercially available Epidian 6 epoxy resin, subjected to the functionalization. An improvement in the adhesion of rubbers to silver wires was observed when using the modified resin. In some cases, an improvement in the mechanical properties of the rubber was observed, especially when the resin was used for crosslinking together with dicumyl peroxide (DCP). Crosslinking synergy between dicumyl peroxide and the modified resin could be observed especially in the case of PO applied for peroxide curing of SBR and NBR.

## 1. Introduction

Nowadays, it has become necessary to use more and more advanced polymer composites in order to meet increasingly demanding applications. For composites containing metallic phases, it becomes crucial to ensure proper adhesion between the continuous and dispersed phases, which can be helped by adding proadhesive systems in the form of modified resins to polymer matrix [1]. The aim of the study was to determine the effect of the addition of epoxy resin containing peroxide group (PO) [2], synthesized previously, on mechanical properties of some synthetic rubbers and their adhesion to silver. Silver wires have been selected as a tester to check the proadhesive potential of the important technical rubbers: styrene-butadiene (SBR), acrylonitrile-butadiene (NBR) and carboxylated acrylonitrile-butadiene (XNBR), to silver nanofillers. Silver nanowires (AgNW) are one of the promising materials that may ensure electrical conductivity and bioactivity of rubber products and further enhancement of their mechanical properties [3]. Their introduction into the rubber matrix makes possible to create effective load sensors based on changes in the electrical conductivity of the composites [4]. Experiments performed with macroscopic silver wire allow for verifying the ability of PO resin to improve adhesion between AgNW and rubber matrix in future rubber nanocomposites. Based on practices commonly applied in engineering, the so-called “brass” bonding method [5], silver plating of the metal surface as an effective method of improving adhesion in rubber-metal joints, should not be excluded. Due to the negative effect of brass plating, manifesting itself in the accelerated rubber ageing process, the most common way to improve the fiber (cord)/polymer boundary interactions, in tires or other technical rubber composites, has become a proadhesive surface treatment of fibers aimed at developing the surface contact or/and modifying their surface composition [6,7]. New methods of preparation and modification of superficial layer and surface of a metal were presented such as: phosphatizing, metal plating with an interlayer of Ni, ZnCo or ZnNi alloys as well as the coating with bifunctional silanes, especially the mixture of bis(trimetoxysilylopropyl)amine with bis(trimetoxysilylopropyl) tetrasulfide. The subject literature contains information on sandblasting [8], shot blasting [9] or phosphating [10], which lead to the formation of lamellar crystals on the surface of the metal, facilitating stress dissipation in the metal/intermediate layer/rubber interface. Often, surface development is also realized by treating the metal surface with radio frequency [11,12,13,14] or microwave [15] plasma, favorably applying a subsequent or simultaneous plasma polymer coating. Zinc plated steel cords were treated with an RF plasma polymerization coating of acetylene or butadiene in order to enhance adhesion to the rubber compounds [11,12]. In [13] carbon fibers were etched by RF plasma and then coated via plasma polymerization in order to enhance their adhesion to vinyl ester resin. The gases utilized for plasma etching were Ar, N_2_ and O_2_, while the monomers used in plasma polymerization coating were acetylene, butadiene and acrylonitrile. Another method involved the thermal evaporation of PPS to form a buffer layer on Cu, followed by plasma treatment with reactive gases such as O_2_, H_2_, and N_2_ on the PPS buffer layer. However, such modifications pose some technological difficulties: they are usually time and cost consuming and often have a negative impact on the environment. The natural alternative seems to be a proadhesive modification of the polymer matrix, which can be not only technologically less complicated but also proecological. The subject literature describes the use of various modifiers: first of all including functional silanes, resorcinol or resorcinol novolacs [16,17,18,19], thermoplastic resins derived from raw wood rosin containing carboxylic acid groups or carboxylic acid ester groups [20], and nonresorcinol functional melamine resins [21], such as adhesives or coupling agents. Improved adhesion of the rubber to the reinforcing materials based on steel cord and textile fibers was obtained by the incorporation of an effective amount of modified novolacs, prepared by simultaneous reaction of polyhydric phenols with aldehydes and unsaturated hydrocarbons at elevated temperature with acid catalysis [16]. A proadhesive effect was also reported for a rubber composition containing silica litharge, and a cobalt salt, when coated on brass plated steel cords and vulcanize. A further improvement in adhesion was obtained by including a resorcinol resin in the composition [17]. Another paper describes the method for adhesion improvement between uncured natural rubber and carbon steels (CS) by the surface modification of the CS with silane coupling agents comprising amino, thiol, glycidoxy, and isocyanate organic functionality [18]. The reaction mechanism among the CS, silane coupling agent, and natural rubber was studied, revealing that adhesion was optimized and cohesive failure achieved when 3-(trimethoxysilyl)propylamine was used to modify the CS. The CS was shown to be directly silanized by the silane coupling agents, but the reactions between the natural rubber and the silane coupling agents was dependent on silane functionality. The invention described in [19] relates a mixture of an urethane-aldehyde resin prepared by condensation of aldehyde, alkyl urethane, and novolac, to a process for the preparation of their mixture, and its application as adhesion promoter in rubber goods. The examples above clearly show that improving the adhesion between AgNW and rubbers is important when it comes to polymer composites, and the application of resins can help in the improvement of polymer–filler interactions. It is also worth noting that some kinds of resins, in addition to improving interphase interactions [22], can also contribute to the cross-linking of the polymer matrix by increasing the crosslinks’ density [23,24] and possibly causing the modification of their structure. The increased content of longer, more elastic or even labile crosslinks reduces interfacial tension [25]. The longer and more elastic bonds, e.g., of hydrogen bonds or ionic cluster nature, created between macromolecules instead of short and stiff covalent C-C ones, are able to reduce interface tension between the metal wire and the rubber matrix. Such modification can be beneficial in terms of the mechanical properties, adhesion and eventually durability of rubber sensors containing silver nanowires, also influencing their conducting behavior, to which the most attention has been paid so far [26]. The question is if polar, monoperoxy derivative of epoxy resin (PO) is capable of this type of modification.

## 2. Materials and Methods 

### 2.1. Materials

#### 2.1.1. Synthesis of Modified Epoxy Resins

**Scheme 1:**
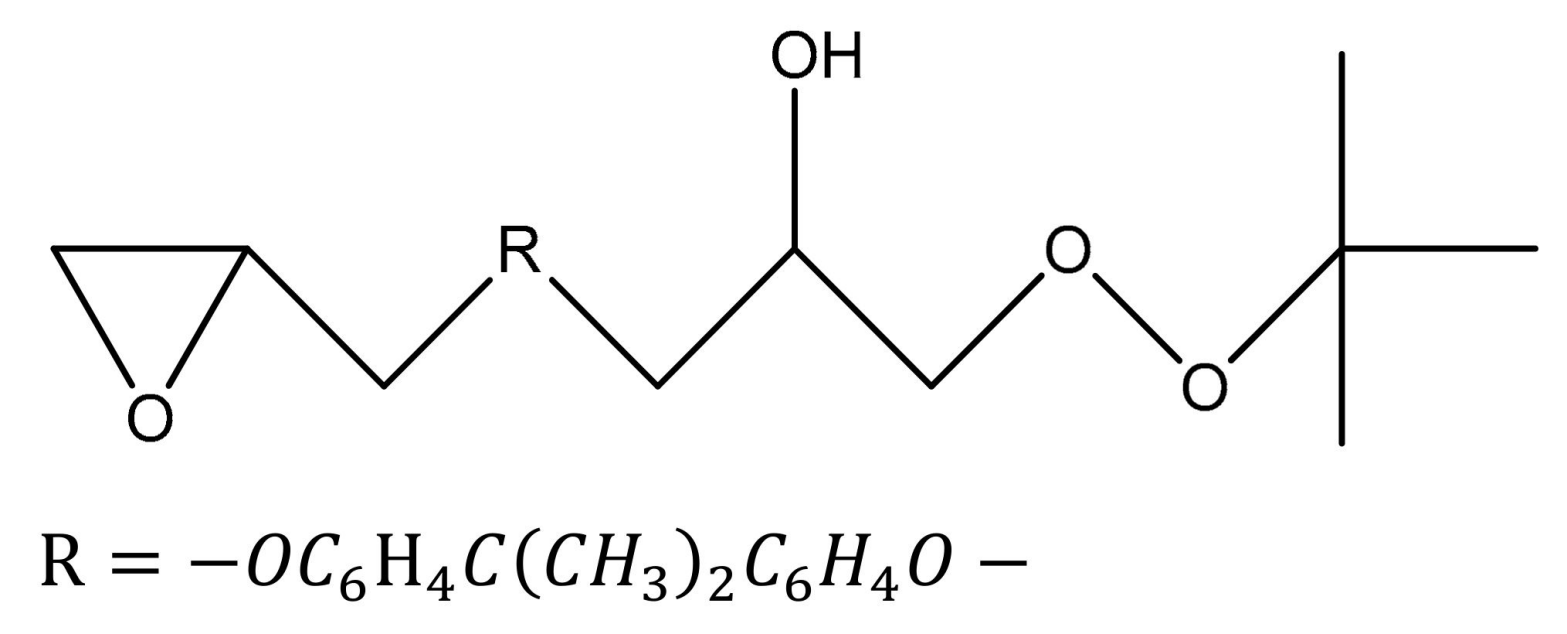
The chemical formula of PO.

Monoperoxy derivative of Epidian 6 epoxy resin (PO) of the formula presented in Scheme 1:

was synthesized in a three-necked reactor equipped with a mechanical stirrer, back condenser, thermometer and dropping funnel. 100 g of Epidian 6 epoxy resin and 100 mL of toluene were placed in the reactor. The mixture was heated with stirring to 50 °C and next, gradually adding the mixture containing 84 g of 70% aqueous tert-butyl hydroperoxide solution, 19.1 g of benzyl triethylammonium chloride (BTEACh) and 3.9 g of potassium hydroxide (KOH), dissolved in 100 mL of toluene. The whole reactor content was kept at 50 °C with stirring for 6 h, then cooled down to room temperature and transferred to the separatory funnel. The aqueous layer was separated, and the organic residue was washed with water until it became neutral. The product was transferred to a vacuum distillation unit, where its distillation was carried out at 50–60 °C and a residual pressure of 133–266 Pa until distilling 2/3 of the flask content was finished. Then the residue was poured into petroleum ether. After separation of the oligomeric product, petroleum ether was drained, and the residue dried under vacuum at 50–55 °C to constant weight. In this way, 120.8 g of PO with 420 g/mol molecular weight, 2.5% of active oxygen and 8.4% of epoxy content was obtained. Physical properties of the resin are presented in our previous paper [2].

#### 2.1.2. Preparation of Rubber Vulcanizates

Composition of the rubber compounds studied is presented in Table 1.

The rubber compounds studied were prepared in a Brabender Plasticorder laboratory micromixer (Germany), and then plate-calibrated to 2 mm thickness. The following mixing conditions were applied: rotational speed of 30 rpm; time of 7 min, room temperature. First, the rubber was plasticized for 3 min, then the appropriate amount of dicumyl peroxide (DCP, 98% of purity, Sigma-Aldrich Ltd., Poznań, Poland) was added and mixed together for another 2 min, and then the PO resin was added to the micromixer chamber. The whole content was then mixed for another 2 min. The samples were crosslinked in a steel mold under pressure, at 160 °C and during the optimum time of curing—t90, determined rheometrically according to ISO 3417.

### 2.2. Methods

#### 2.2.1. Kinetics of Crosslinking

The curing process of rubber mixes was investigated using an Alpha MDR 2000 (USA) rheometer, operating with an oscillation frequency of 1.70 Hz and an oscillation angle of 3°, at 160 °C, according to ISO 3417. Based on experimental data, the curing parameters were determined, namely: optimum vulcanization time (t90), scorch time (t05), max. (MH), and min. (ML) torque. The increase of torque ΔM = MH − ML

#### 2.2.2. Mechanisms of Crosslinking

The mechanisms of rubber crosslinking was studied with Fourier Transformed Infrared Spectroscopy (FTIR). Absorbance spectra were collected within the 400–4000 cm^−1^ range (64 scans, resolution of 2 cm^−1^) and the characteristic absorption peaks assigned. Experiments were performed with a Thermo Fisher Scientific (USA) Nicolet 6700 FTIR spectrometer equipped with a Harrick Scientific (USA) SplitPea micro ATR accessory with a diamond crystal.

#### 2.2.3. Crosslink Density of Rubber

Crosslink density of the rubber samples (ν) was determined by equilibrium swelling method in toluene, according to PN-ISO 1817: 2001. Samples cut from 2 mm thick plates, weighing 30–50 mg, were exposed to the solvent for 72 h, subjected to decanting and replenishing every 24 h. After drying on a filter paper, the samples were weighed again and then dried to constant weight at 60 °C in a laboratory oven. The results obtained were used to calculate volumetric equilibrium swelling values of the rubber samples, according to the standard procedure described in the literature [27], and their crosslinking density—applying calculated values of polymer—toluene Flory–Huggins′ parameters [28] and using the Flory–Rehner equation [29].

#### 2.2.4. Mechanical Properties of Rubber

Tensile Strength (TS) of crosslinked rubber samples was determined with an universal mechanical testing machine Zwick 1345 (ZwickRoell GmbH & Co. KG, Ulm, Germany), according to ISO 37. Tear Strength (TES) of the samples was determined with the same instrument—Zwick 1345 (ZwickRoell GmbH & Co. KG, Ulm, Germany), according to ISO 34. Hardness of the samples was determined with a Zwick Shore A durometer 3130 (ZwickRoell GmbH & Co. KG, Ulm, Germany), according to EN ISO 868.

#### 2.2.5. Adhesion

**Figure 1:**
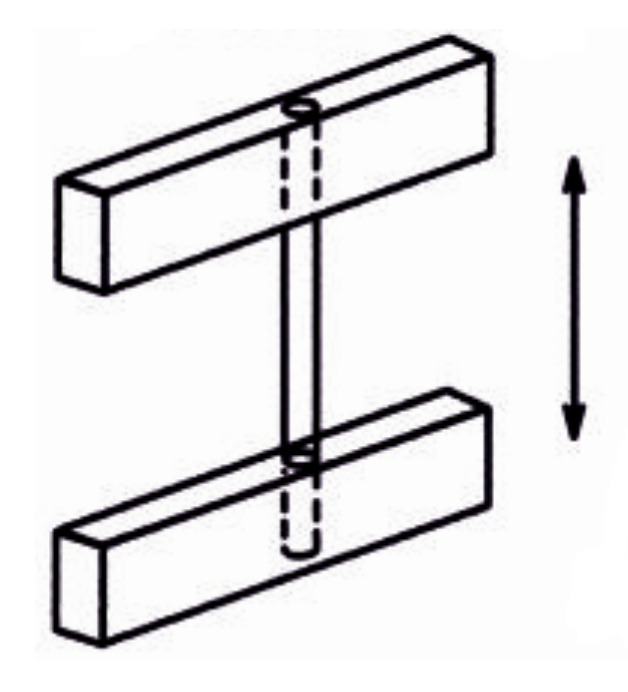
Illustration of the “H” type specimen. A silver wire fixed in two rubber plates during their crosslinking.

The pull-out tests of silver wires (ø = 0.5 mm) from crosslinked rubber samples were performed according to PN-81/C-04267 (the so-called method of the “H” type specimen, see Figure 1, using an universal mechanical testing machine Zwick 1345 (ZwickRoell GmbH & Co. KG, Ulm, Germany), operating with a linear speed of 50 mm/min. 

## 3. Results

### 3.1. Kinetics of Crosslinking

**Figure 2:**
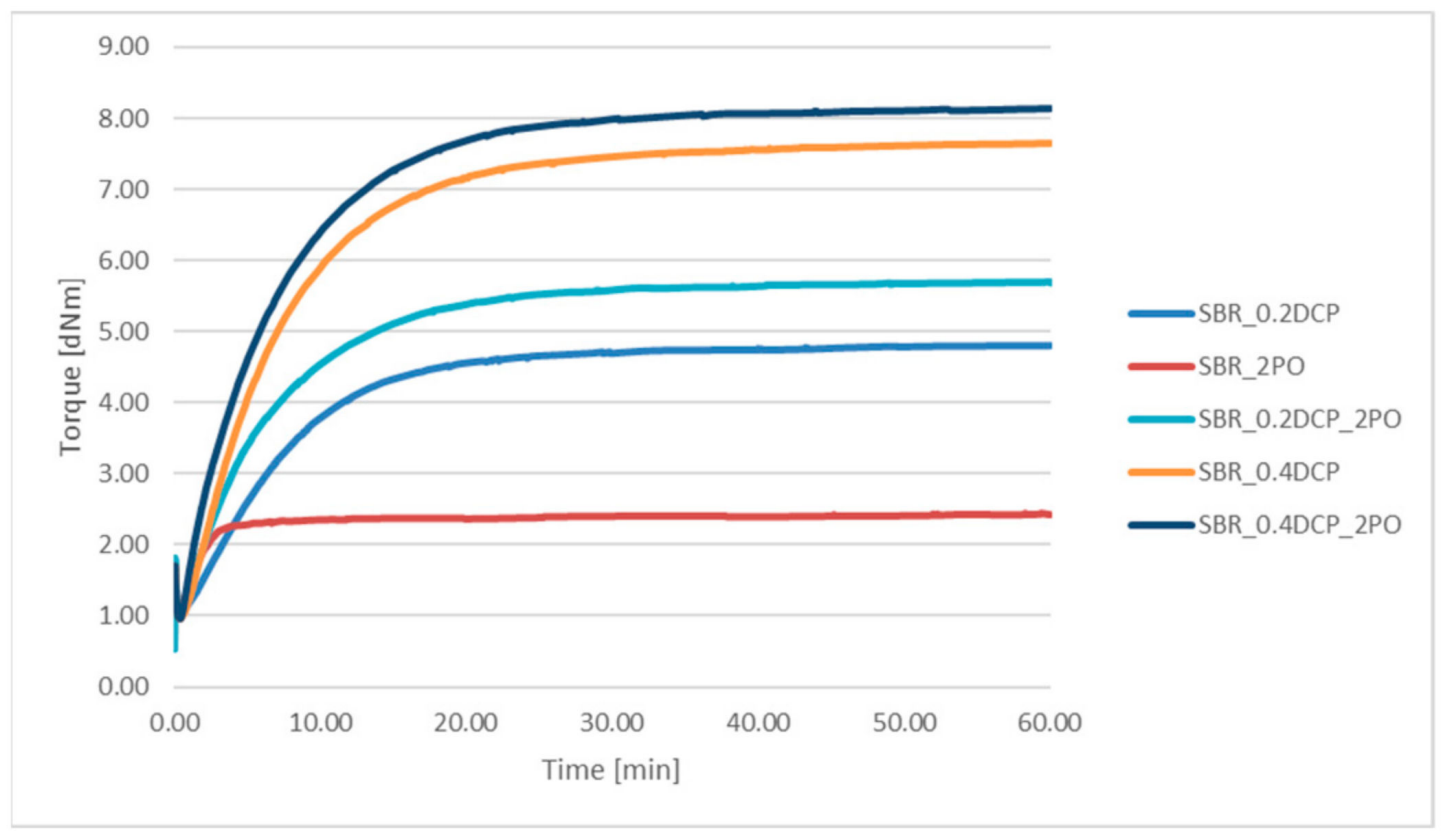
Crosslinking kinetics of the compounds based on styrene-butadiene rubber (SBR).

**Figure 3:**
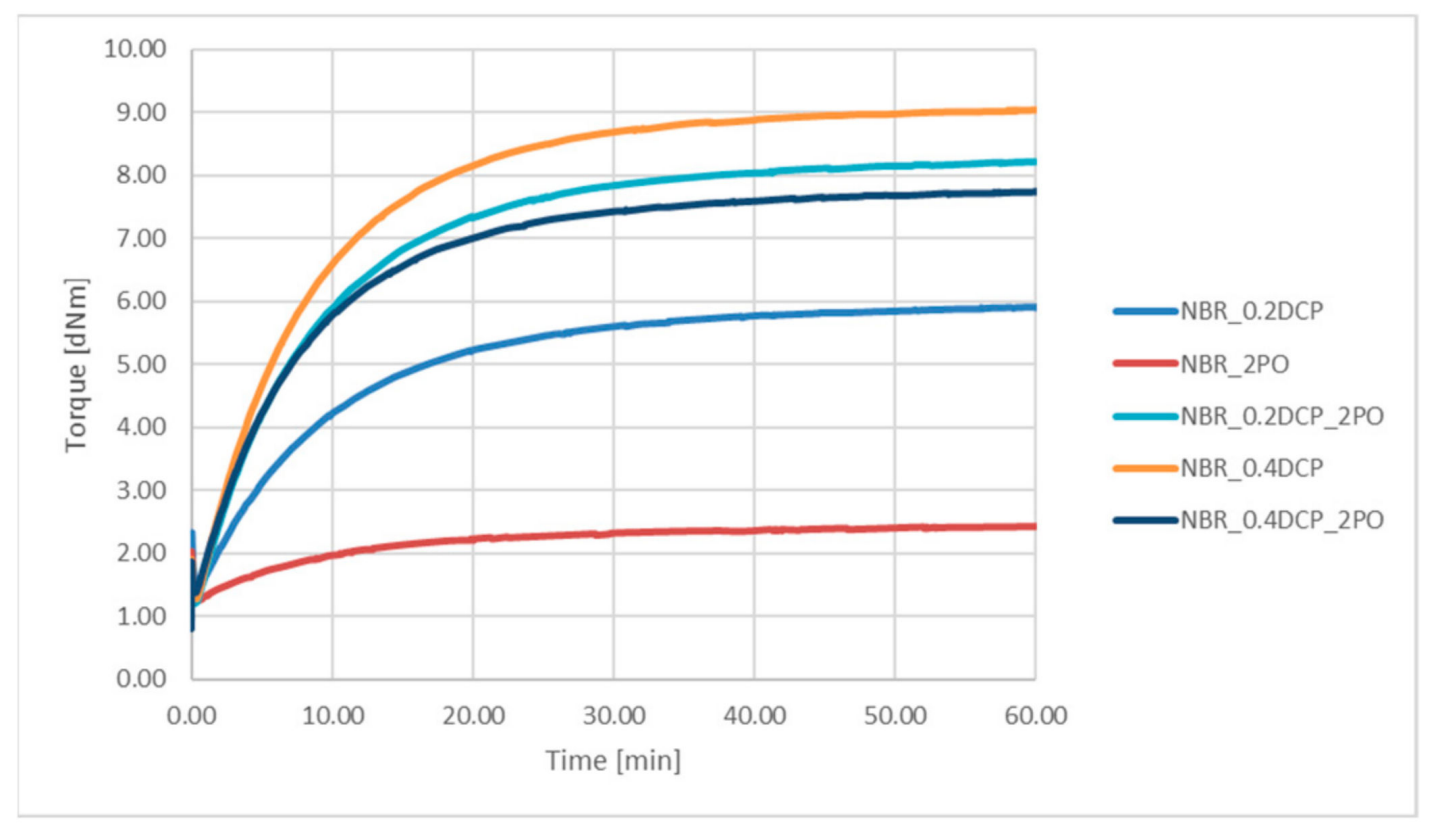
Crosslinking kinetics of the compounds based on acrylonitrile-butadiene rubber (NBR).

**Figure 4:**
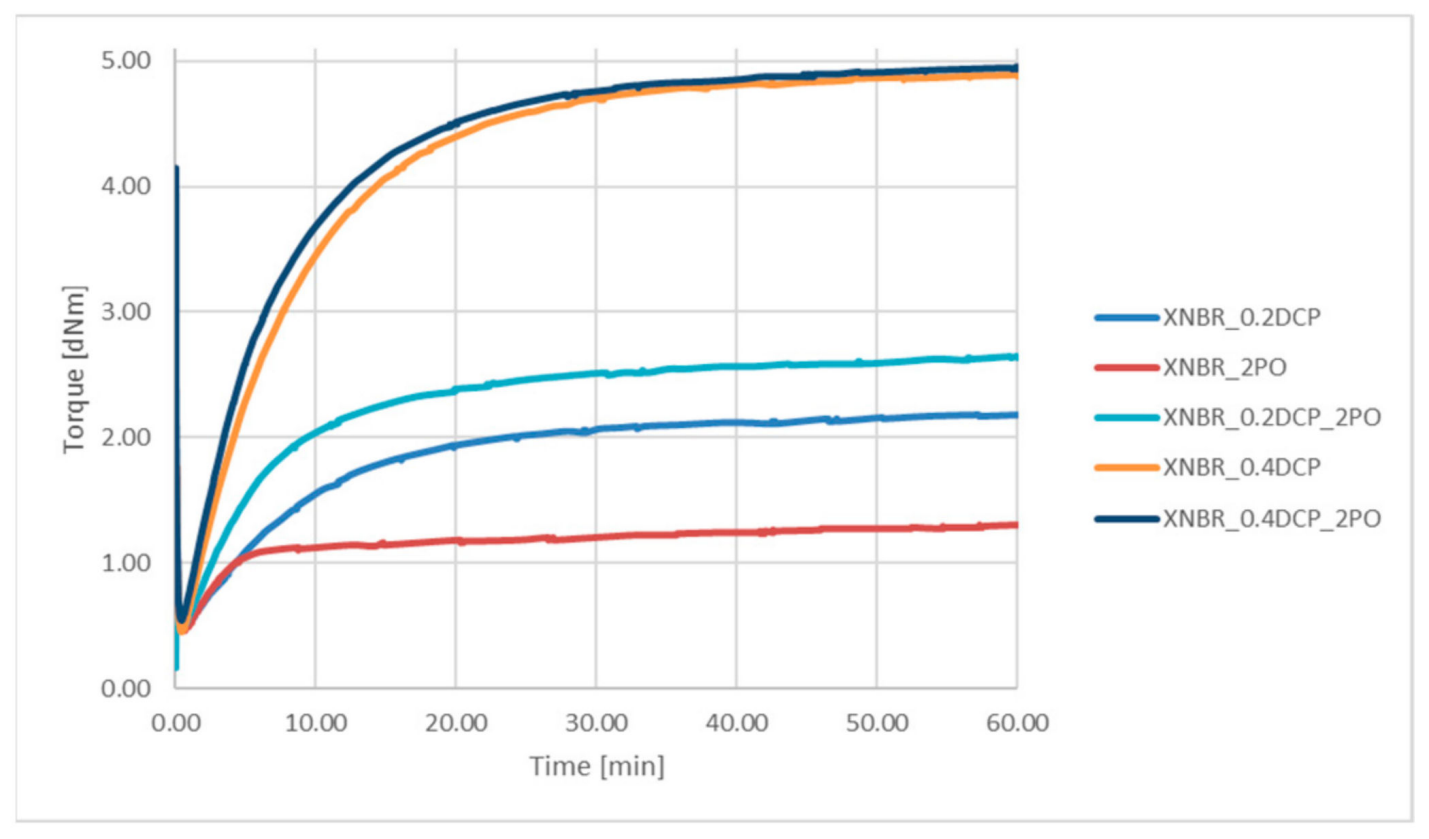
Crosslinking kinetics of the compounds based on carboxylated acrylonitrile-butadiene rubber (XNBR).

Crosslinking curves of the rubber compounds tested are presented in Figure 2, Figure 3 and Figure 4, while the curing parameters are summarized in Table 2.

The values collected in Table 2 are the conventional parameters of vulcanization, recommending optimum vulcanization time (t90) and time of safety processing (t05) at a given temperature. Modifying rubbers by a resin addition we consider it advisable to check how this will affect the vulcanization parameters. They are not directly related to the adhesion of the rubbers to silver, but if the addition of PO makes the crosslink density of the rubber high enough, it can be responsible for very weak adhesion to silver due to the different thermal shrinkage of the metal and the rubber after vulcanization (see an example of highly crosslinked NBR). The analysis of the rheometric curves confirms the limited ability of the modified PO resin to crosslink the tested rubbers on its own. However, the situation changes if it is used as a coagent of peroxide crosslinking in the presence of DCP. The effect, expressed by an increase of torque, is most visible in the case of SBR-based compounds, whereas it is visible to a lesser extent for NBR- or XNBR-based mixes. ΔM is the largest if the resin is added to a system containing 0.2 phr of DCP. Further increase of peroxide content no longer makes MH increased. No effect of modified resin addition on the curing time of SBR-based compounds is observed. Short vulcanization time—t90, for the SBR_2PO system—is a consequence of the low MH value obtained by the sample. However, in the case of mixes based on NBR and XNBR, a slight reduction in the curing time is seen as a result of introducing the resin to the DCP crosslinking system. The addition of the modified resin has practically no effect on the curing time of the rubber compounds tested. A shorter scorch time—t_05_ is only characteristic for SBR mixes containing 2 phr of the resin, being accompanied by a slight increase in torque. Other compounds exhibit the same level of processing safety as those crosslinked with peroxide. There are no significant differences in the minimum torque values of the rubber compounds tested. However, for the mixes based on XNBR, it is clearly lower in comparison to the others.

### 3.2. Crosslink Density of Rubber

**Figure 5:**
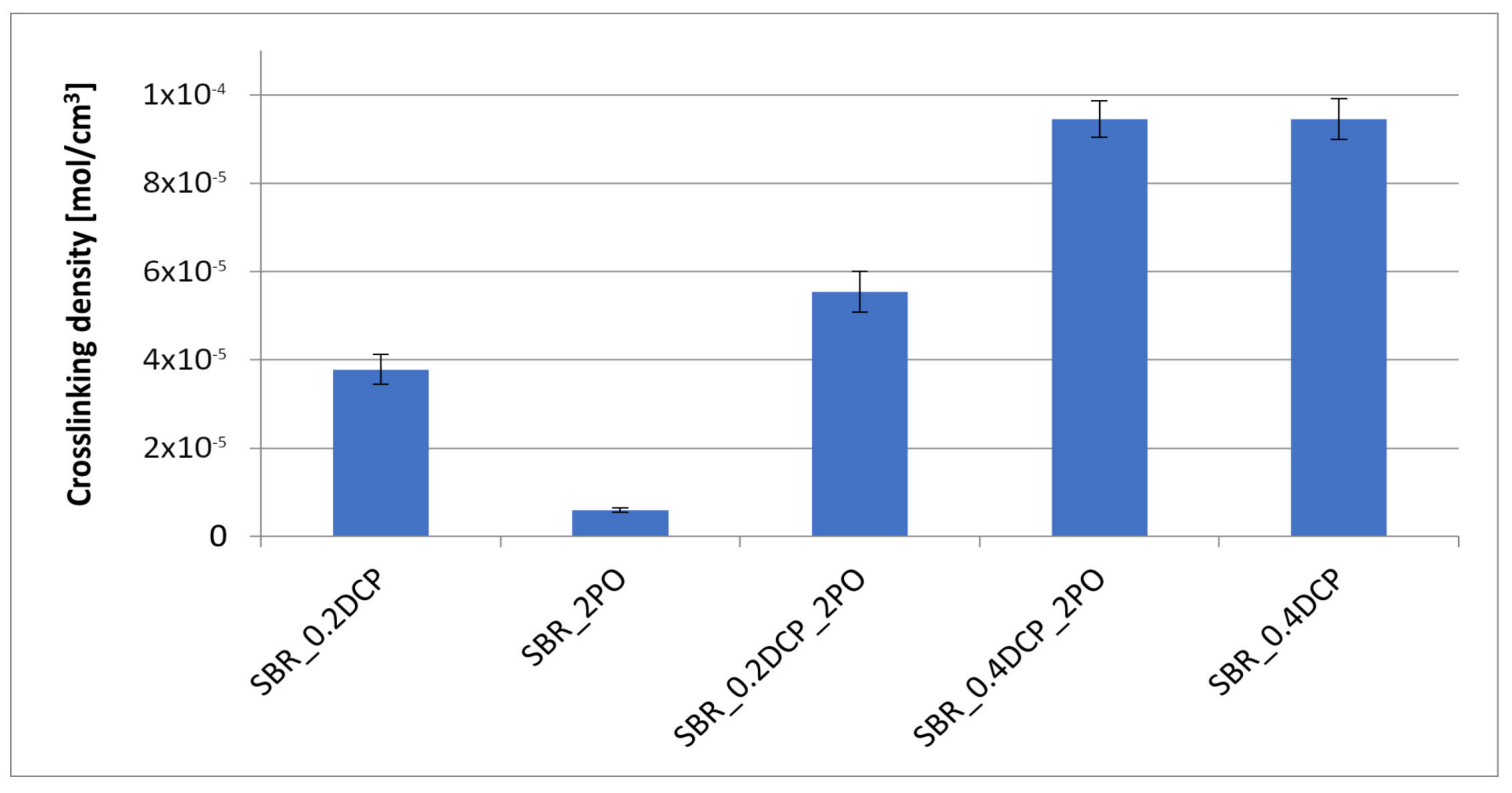
Influence of the modified resin addition on the crosslink density of SBR.

**Figure 6:**
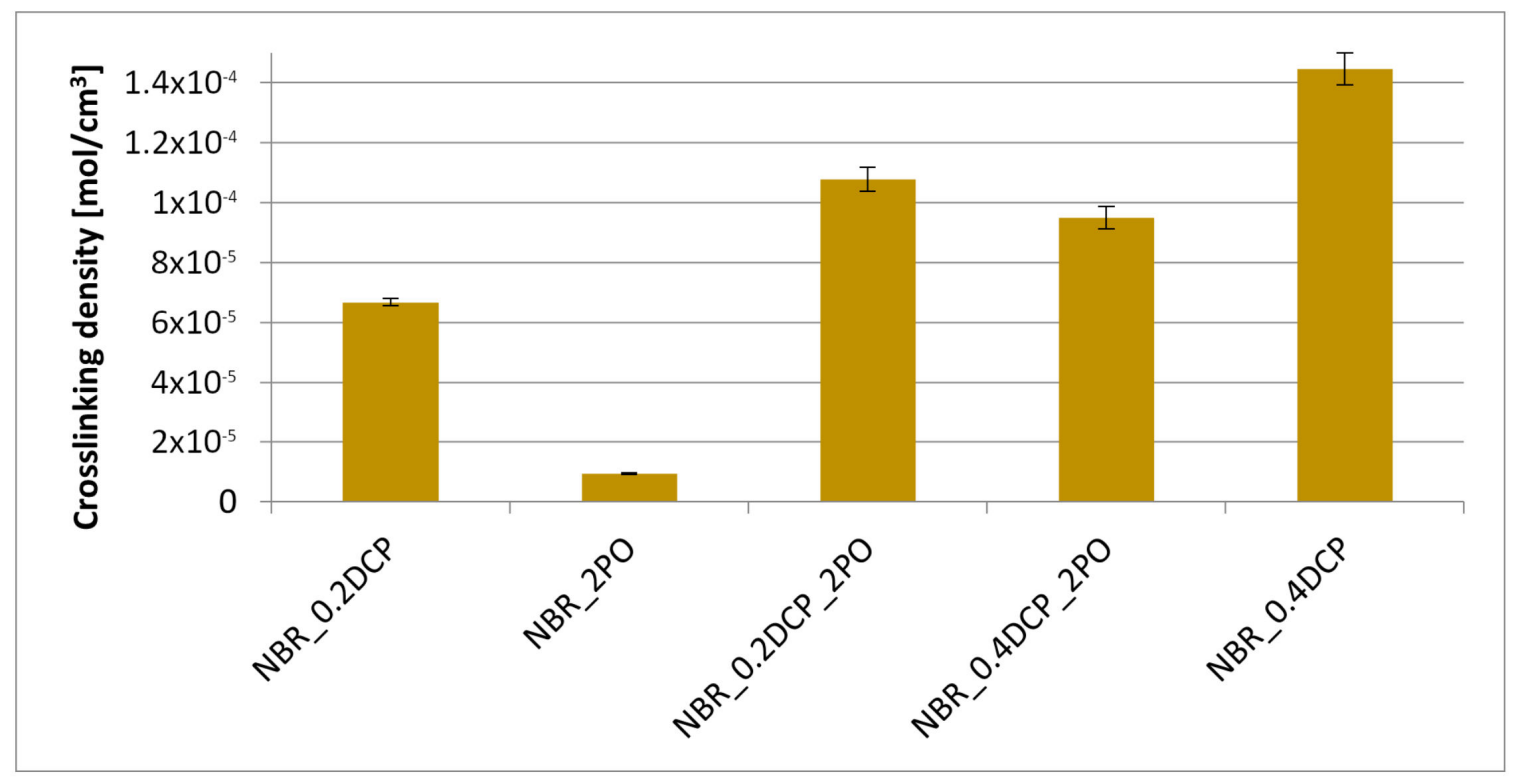
Influence of the modified resin addition on the crosslink density of NBR.

**Figure 7:**
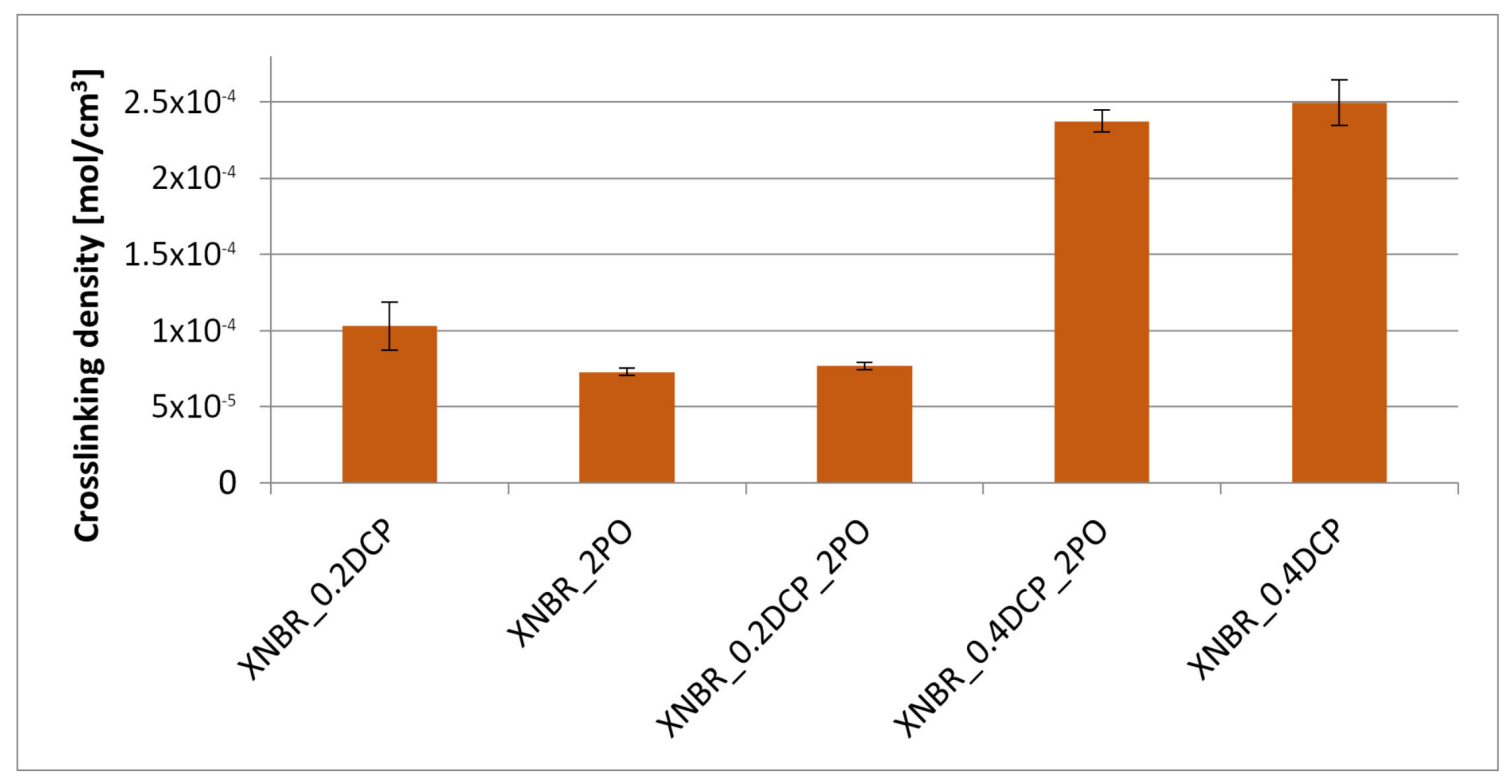
Influence of the modified resin addition on the crosslink density of XNBR.

The comparison of the crosslink density of the cured rubbers tested, determined based on their equilibrium swelling in toluene, is presented for each of the rubbers in Figure 5, Figure 6 and Figure 7.

The results of the equilibrium swelling of the cured samples in toluene do not match the increase of their rheometric torque. This fact may suggest a post-curing-specific interaction of hydrogen bond character between the rubber macromolecules, which are not stable at the crosslinking temperature (160 °C). It is particularly visible in the case of the XNBR samples, which clearly exceed the crosslink density of the SBR and NBR rubbers. Only XNBR could be cured with the resin, even without any addition of DCP. The curing of SBR and NBR is possible with PO resin alone, but the crosslink density values obtained are very low. 

Compared to SBR, both nitrile rubbers achieve higher crosslinking densities using DCP, because, unlike the former, they are additionally capable of thermal crosslinking (NBR) [30], or specific interactions of hydrogen bond nature or ionic cluster formation (XNBR) [31]. However, the synergy of the crosslinking action of the modified resins and DCP can only be confirmed for SBR and NBR.

The suggested mechanisms of rubber crosslinking with DCP and the modified resin are presented below:

#### 3.2.1. Styrene-Butadiene Rubber (SBR)

**Scheme 2:**
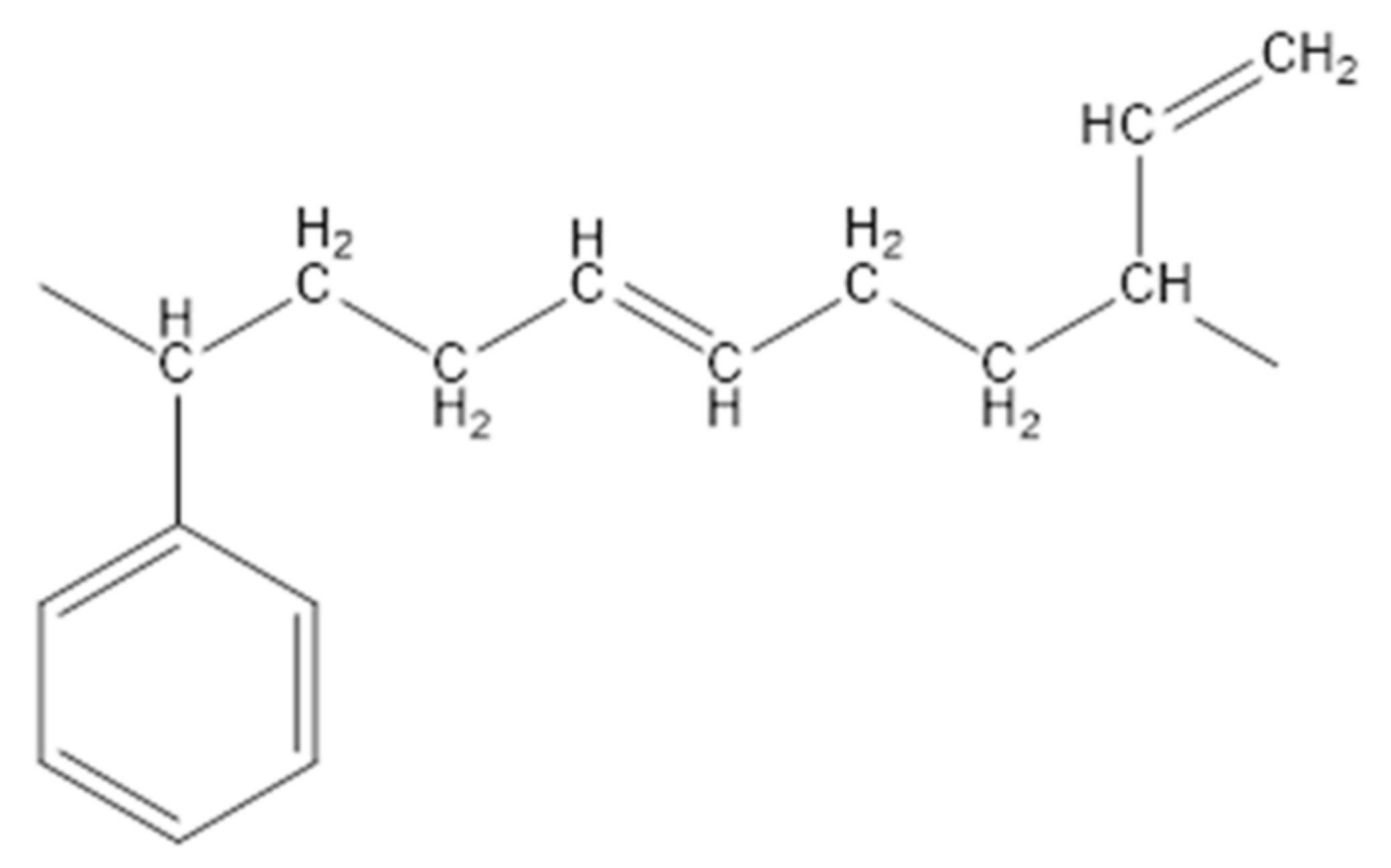
The general structure of SBR.

**Scheme 3:**
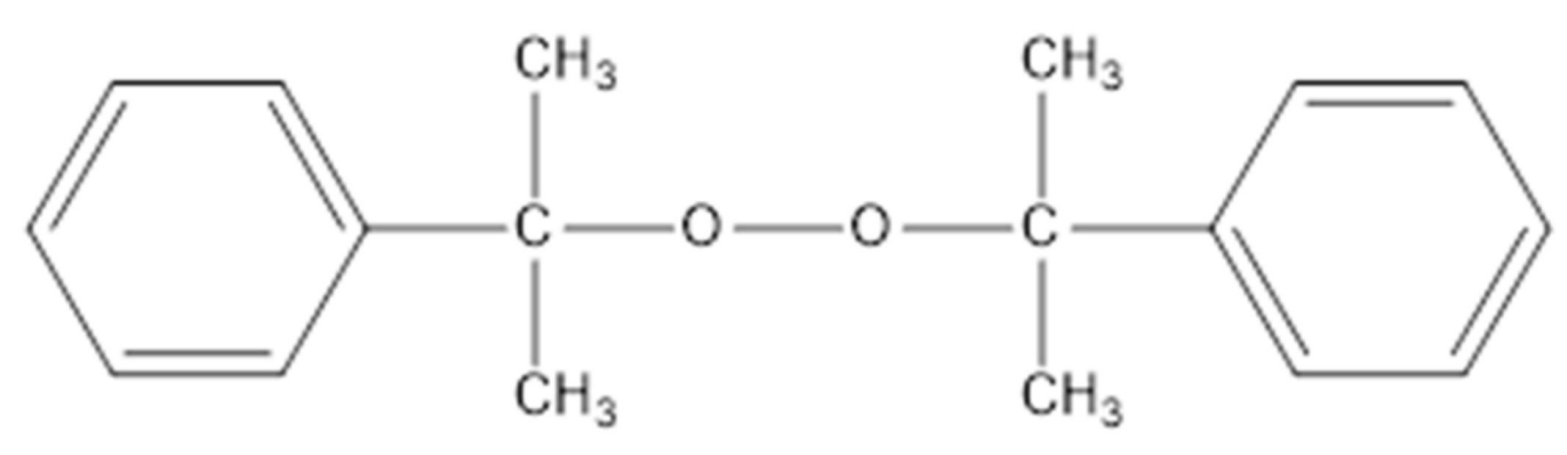
Formula of DCP.

Crosslinking of SBR—Scheme 2 using dicumyl peroxide (DCP)—Scheme 3, is presented below [32]:

(1)
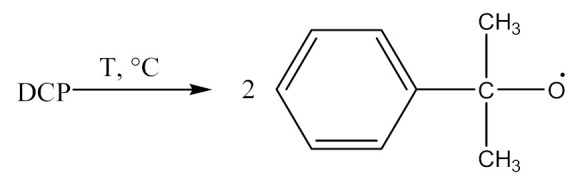



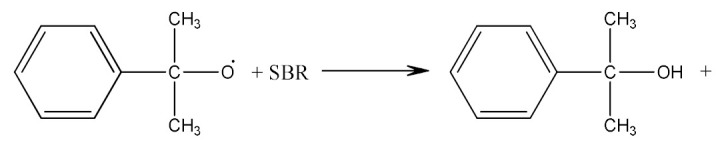


(2)
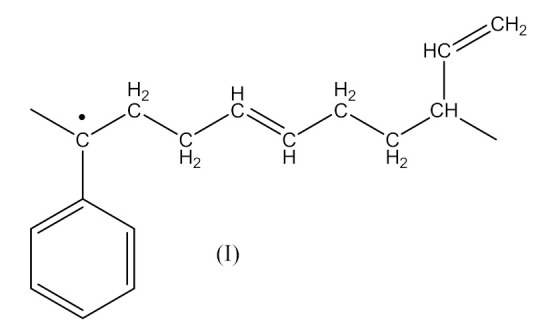


(3)
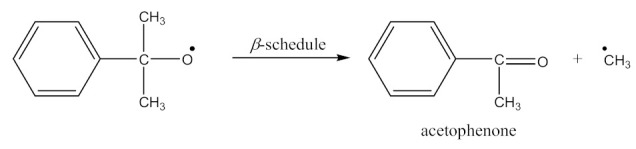


(4)
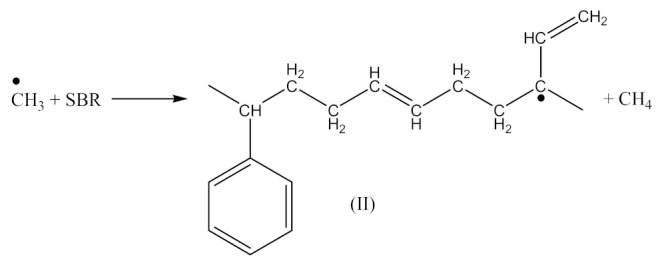


(5)
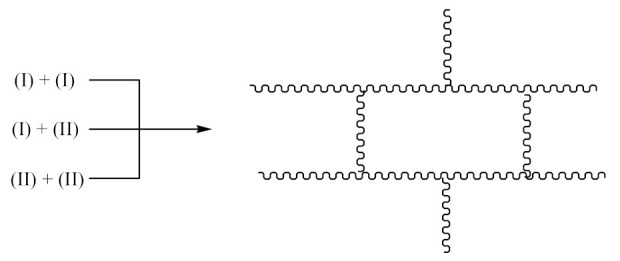


Crosslinking of SBR using PO:

(6)
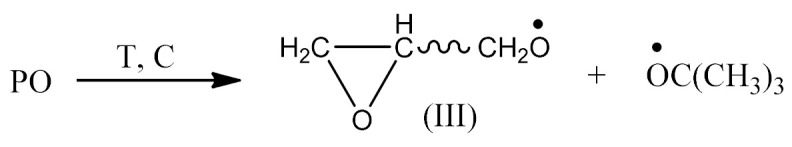


(7)
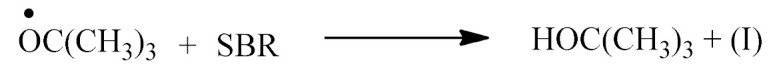


(8)
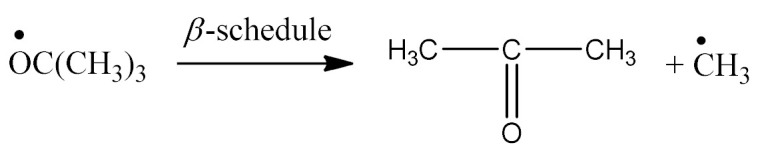


(9)
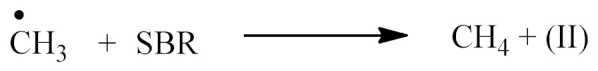


(10)
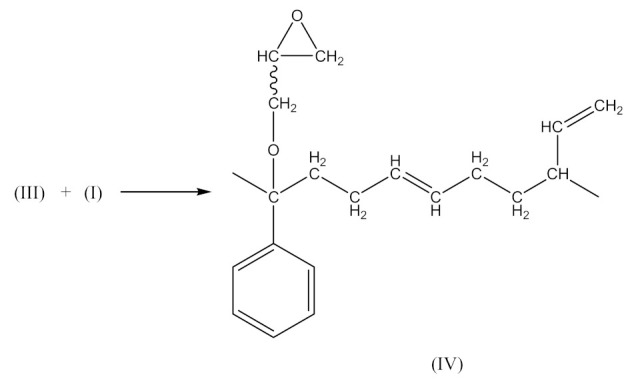


(11)
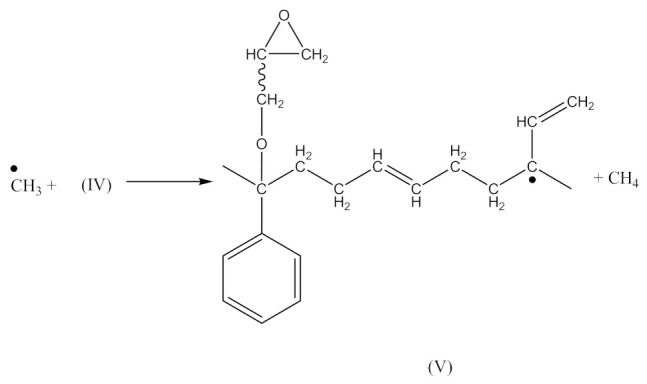


(12)
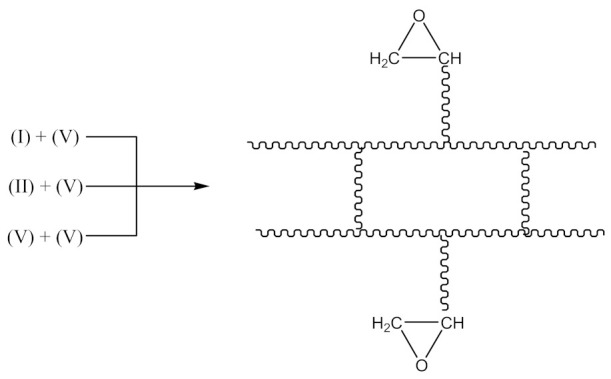


**Figure 8:**
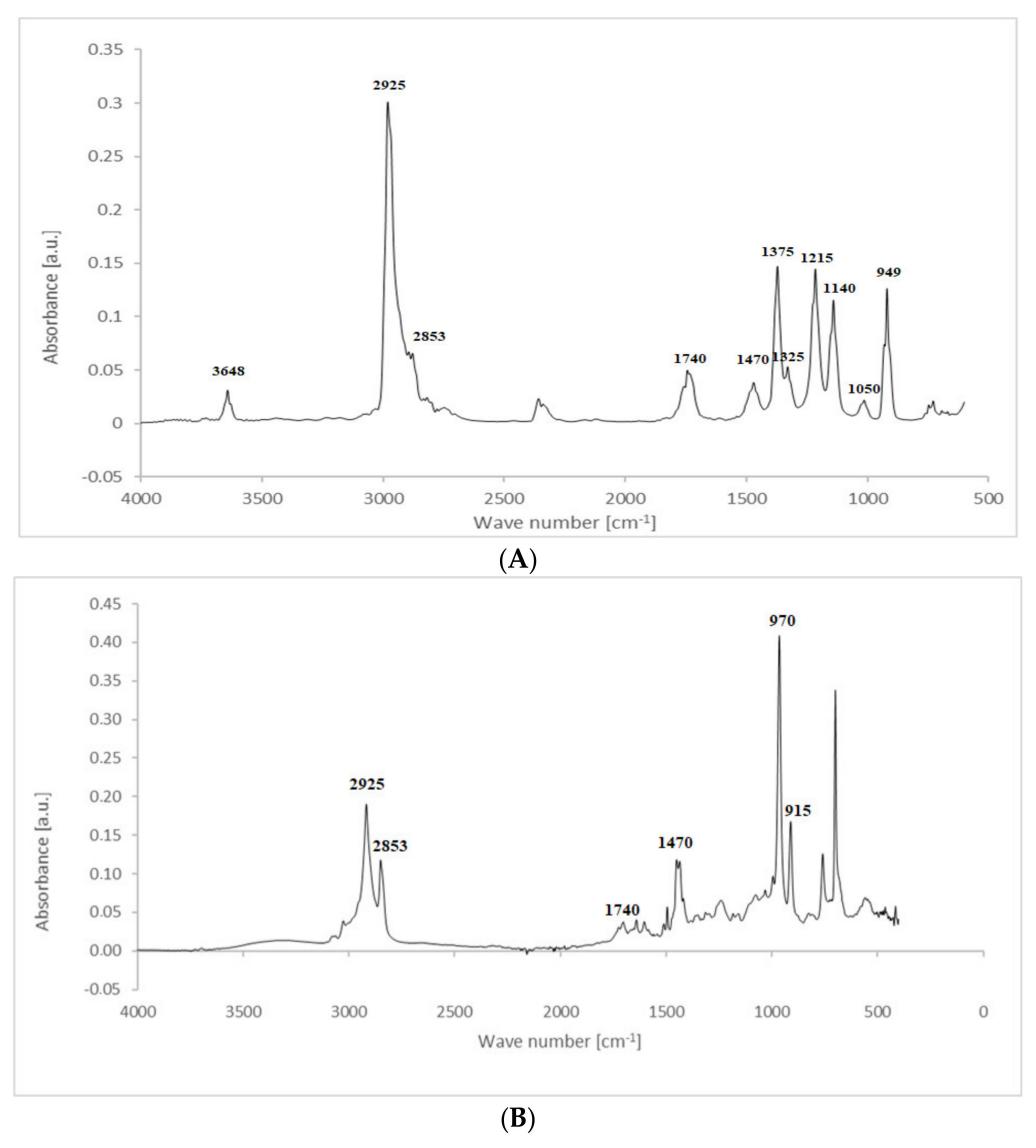
FTIR spectrum of SBR crosslinked with PO resin: (**A**) PO resin, (**B**) SBR_2PO.

FTIR spectrum of PO crosslinked at 160 °C (Figure 8A) contains characteristic absorption peaks at:

- 3648 cm^−1^, vibrations of free OH groups
- 2925 and 2853 cm^−1^, symmetric and asymmetric vibrations of CH_2_ and CH_3_ groups
- 1740 cm^−1^, related to C = O vibrations, probably originated from aldehyde groups,
- 1470 and 1375 cm^−1^, vibrations of CH_2_ and CH_3_ groups
- 1325, 1215, 1140 and 1050 cm^−1^, vibrations in alcohol and/or ether fragments,
- 949 and 912 cm^−1^ vibrations characteristic for epoxide groups.

Monoperoxy derivative of Epidian 6 epoxy resin (PO) contains free epoxy, secondary hydroxy and labile peroxy groups in its structure. Due to the peroxide curing of SBR, the decomposition of –O–O– bond in PO occurs, and free radicals are formed as is described in Equation (6). The formed (CH_3_)_3_CO• radical is able to attack the SBR molecule according to Equation (7) and turns into tert-butyl alcohol or may form an acetone molecule and CH_3_• radical via the β-schedule. The CH_3_• radical can further attack the rubber molecule, which is described in Equation (9). The oligomer radical (III), (Equation (6)) undergoes a recombination reaction (Equation (10)) with radicals formed according to Equations (7) and (9), and thus becomes attached to SBR molecule. As a result, the SBR molecule contains free epoxy and secondary hydroxy groups. IR spectroscopic studies of SBR_PO after curing were conducted in order to confirm the abovementioned reactions. The absorption band at 949 cm^−1^, corresponding to the stretching vibrations of the epoxy ring, is observed in the FTIR spectrum (Figure 8).

The absorption peaks at 2830 and 2745 cm^−1^, associated with CHO group vibrations in aldehyde, confirm the formation of aldehyde in the decomposition products of the resin. The above peaks, together with a small but characteristic absorption peak at 3016 cm^−1^, associated with methane particles, arising in FTIR spectra at higher temperature, confirm the proposed mechanism of the thermal decomposition of PO resin (Equations (6)–(8)). 

In the FTIR spectrum of the crosslinked rubber (Figure 8B), apart from the absorption peaks characteristic for SBR, one can notice arising peaks at 970 and 915 cm^−1^, and the lack of peaks from aldehyde, which confirms the proposed mechanism of crosslinking SBR with PO presented by Equations (10) and (11). 

The crosslinking of SBR using DCP + PO proceeds according to Equations (1)–(12).

#### 3.2.2. Acrylonitrile-Butadiene Rubber (NBR)

**Scheme 4:**
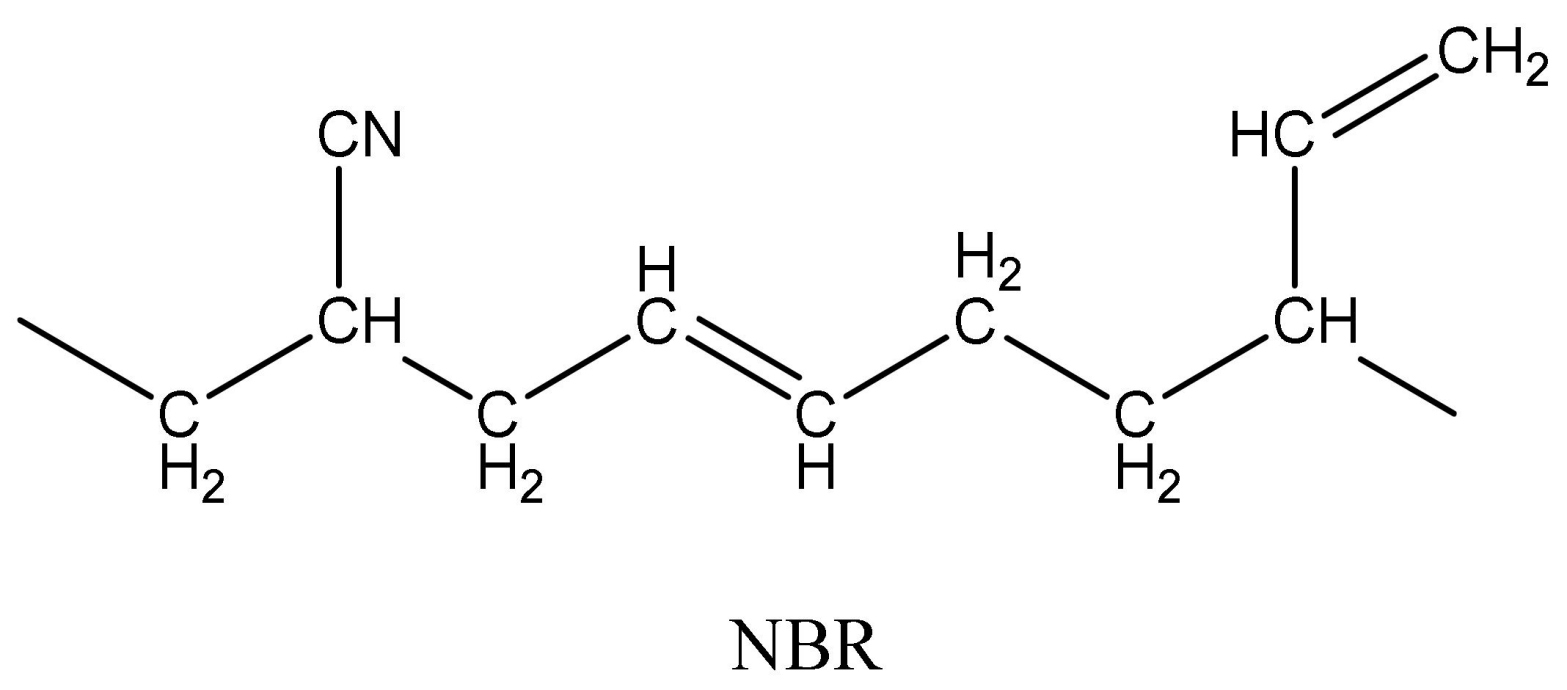
The general structure of NBR.

Crosslinking of NBR—Scheme 4 using DCP proceeds according to Equations (1)–(4).

The radical formed by Equation (2) has the form:


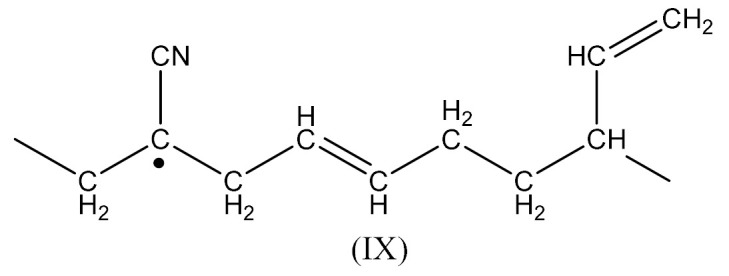


Whereas the radical formed by Equation (4) has the form:


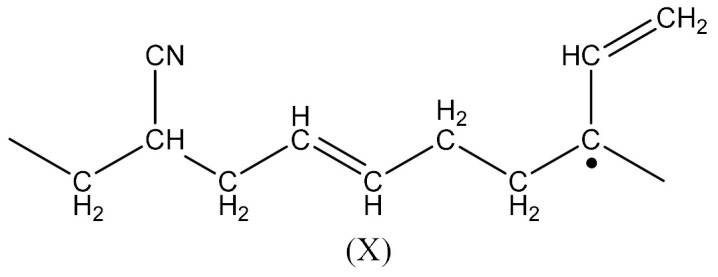


During curing by DCP, crosslinking occurs as a result of the interaction:

(13)
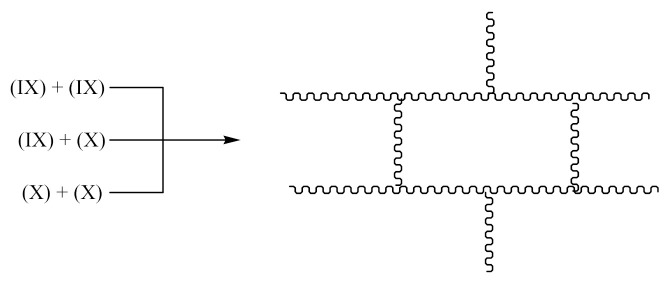


Crosslinking of NBR using PO:

In this case, there are reactions that should occur by the proposed Equations (6)–(11). Radicals formed according to Equations (7) and (9) look like the radical (IX) and (X), respectively. When the radical (III) is reacted with (IX), a compound of the structure shown below is the most likely formed:


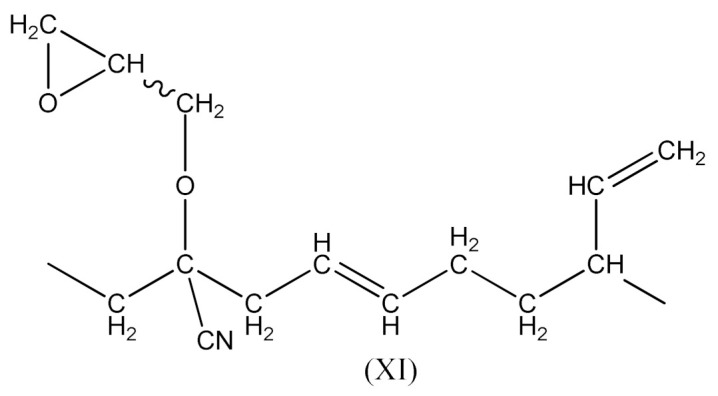


When CH3· radical interacts with (XI), a new radical is formed (XII):


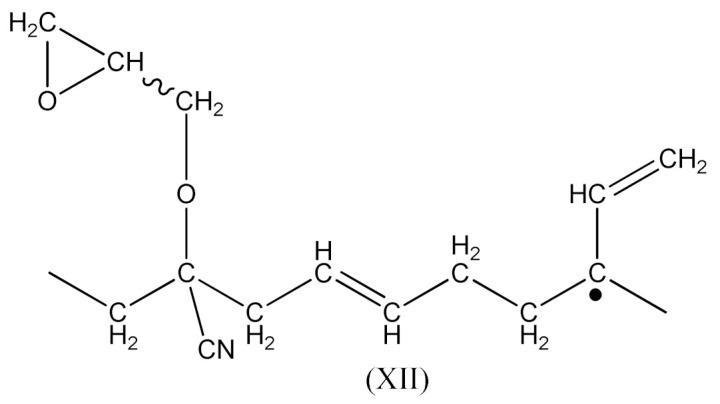


Then the NBR vulcanization involving the PO occurs as a result of the interaction:

(14)
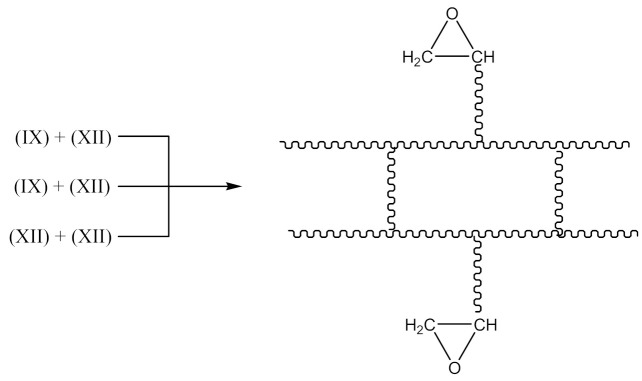


The absorption peak at 1040 cm^−1^ proves the presence of secondary hydroxy groups. The peaks at 1100 and 1239 cm^−1^ corresponding to –C–O–C– asymmetric stretching vibrations indicate the attachment of the PO fragment to the SBR molecule. The introduction of the PO fragment into the SBR structure is also confirmed by an absorption peak at 758 cm^−1^, corresponding to –C–H deformation stretching vibrations in 1,4-disubstituted benzene derivatives. Absorption peaks at 698, 963, 2849 and 2916 cm^−1^ corresponding to the deformation stretching vibrations of –C–H in styrene, nonplanar deformation vibrations of –C–H in–C=C–H, symmetric and asymmetric stretching vibrations of CH2 in saturated hydrocarbons, respectively, prove the presence of SBR fragments. The occurrence of curing (in accordance with Equations (10) and (11)) and the preservation o thef double bond is confirmed by absorption peaks at 963, 1449 and 1639 cm^−1^, corresponding to the –C=C–deformation stretching vibrations.

**Figure 9:**
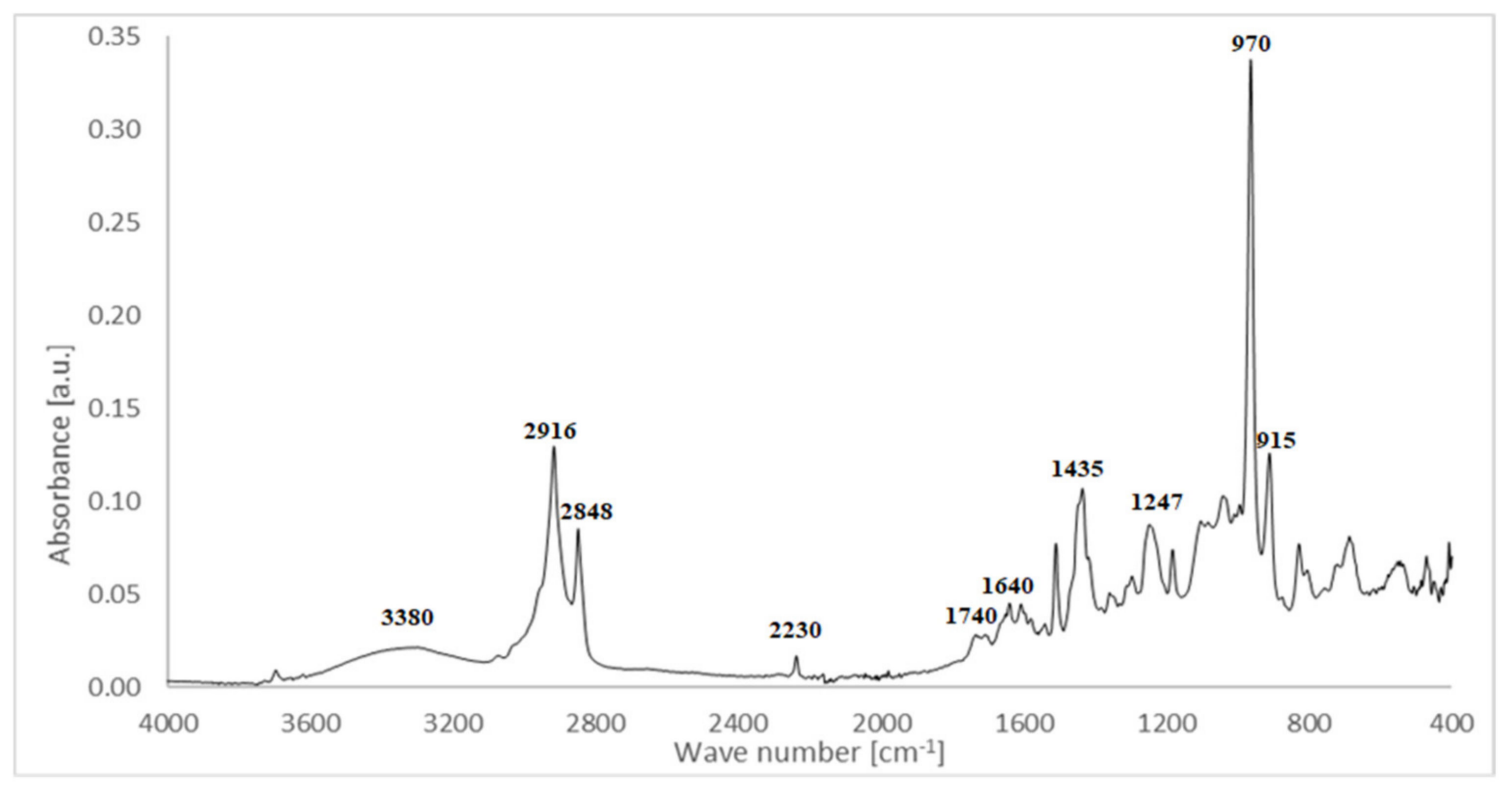
FTIR spectrum of NBR crosslinked with PO resin (NBR_2PO).

The analysis of the IR-spectrum of the NBR_2PO (Figure 9) shows the presence of free epoxy groups (band at 912 cm^−1^) and secondary hydroxy groups (peaks at 1042, 1183 and 3380 cm^−1^). The structure XII is formed in the same manner as described above and is confirmed by –C–O–C–asymmetric stretching vibrations at 1247 cm^−1^. The presence of –C≡N group in NBR is proved by stretching vibrations at 2230 cm^−1^ and the rubber fragment presence —by absorption peaks at 964, 1435, 1640, 2848 and 2916 cm^−1^. Similar to the crosslinking of SBR, in the FTIR spectrum of NBR cured with PO (Figure 9) absorption also peaks at 1740 cm^−1^, indicating that the thermal decomposition products of the resin (Equations (6)–(8)) disappear from the spectrum of the crosslinked rubber. The characteristic absorption peaks originating from C-H bending vibrations in the double pendant bonds at 970 cm^−1^ and 915 cm^−1^ arise, confirming the proposed mechanism of crosslinking NBR with PO resin.

The crosslinking of NBR using DCP + PO proceeds via the reactions proposed by Equations (13) and (14).

#### 3.2.3. Carboxylated Acrylonitrile-Butadiene Rubber (XNBR)

**Scheme 5:**
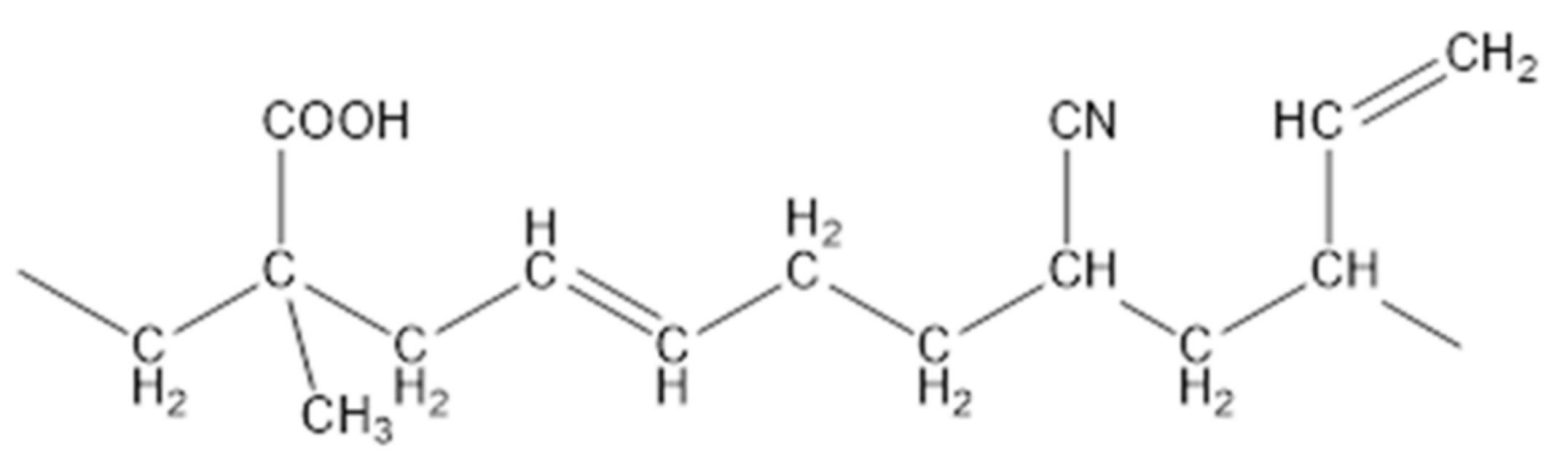
The general structure of XNBR.

Crosslinking of XNBR—Scheme 5 using DCP:

The reaction occurs similarly to Equations (1)–(4). Two possible macroradicals can be formed. Similarly to Equation (2) of the appearance:


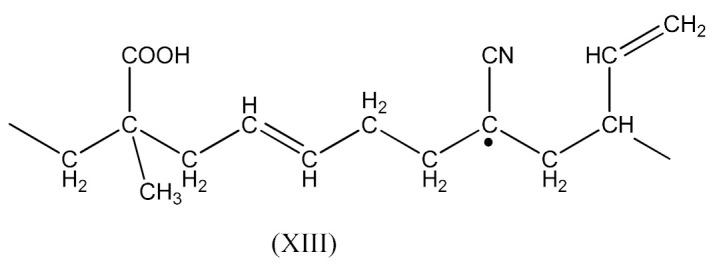


as well as similarly to Equation (4) in the form:


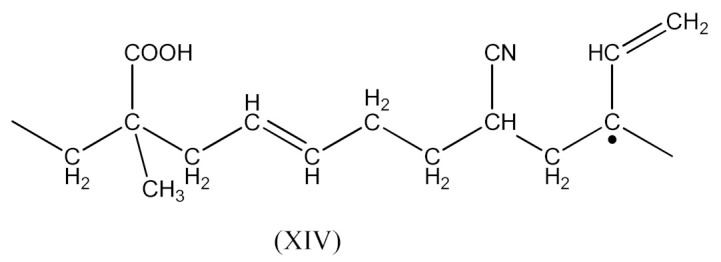


Then the crosslinked structure is formed by the interaction:

(15)
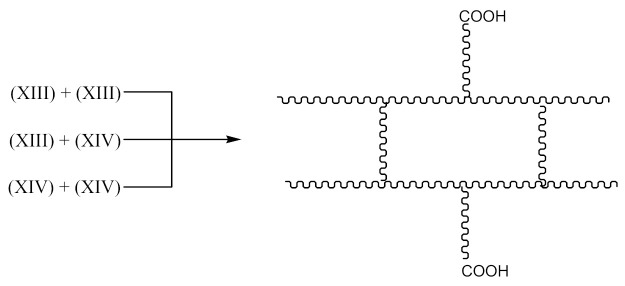


Crosslinking of XNBR using PO:

The reaction occurs similarly to the course described by Equations (6)–(11). Only Equation (7) doubles the radical (XIII), and radical (XIV) is produced according to Equation (9).

A compound of the form below is produced according to Equation (10):


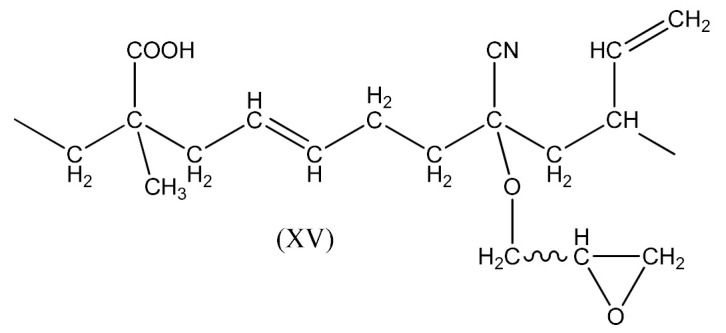


And when CH3· radical interacts with (XV), a radical (XVI) is formed according to Equation (11):


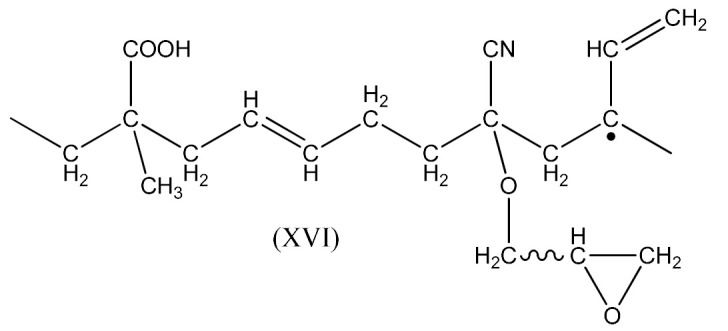


The XNBR fragment presented (XV) and the radical formed (XVI) can react with PO due to the interaction of the free carboxyl group in the above compounds and the epoxy group radical (16):

(16)
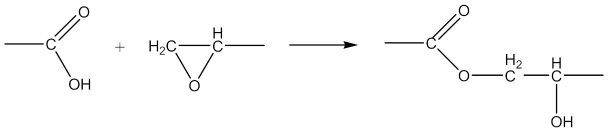


This process can lead to a denser crosslinking. The reaction in this case occurs according to the schemes below Equation (17):

(17)
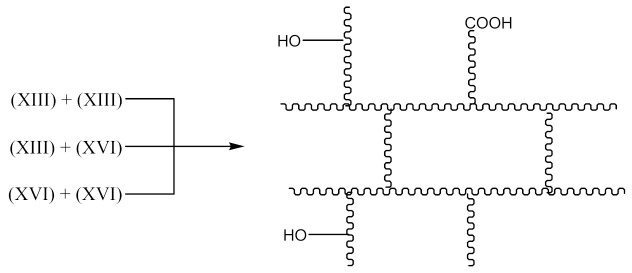


**Figure 10:**
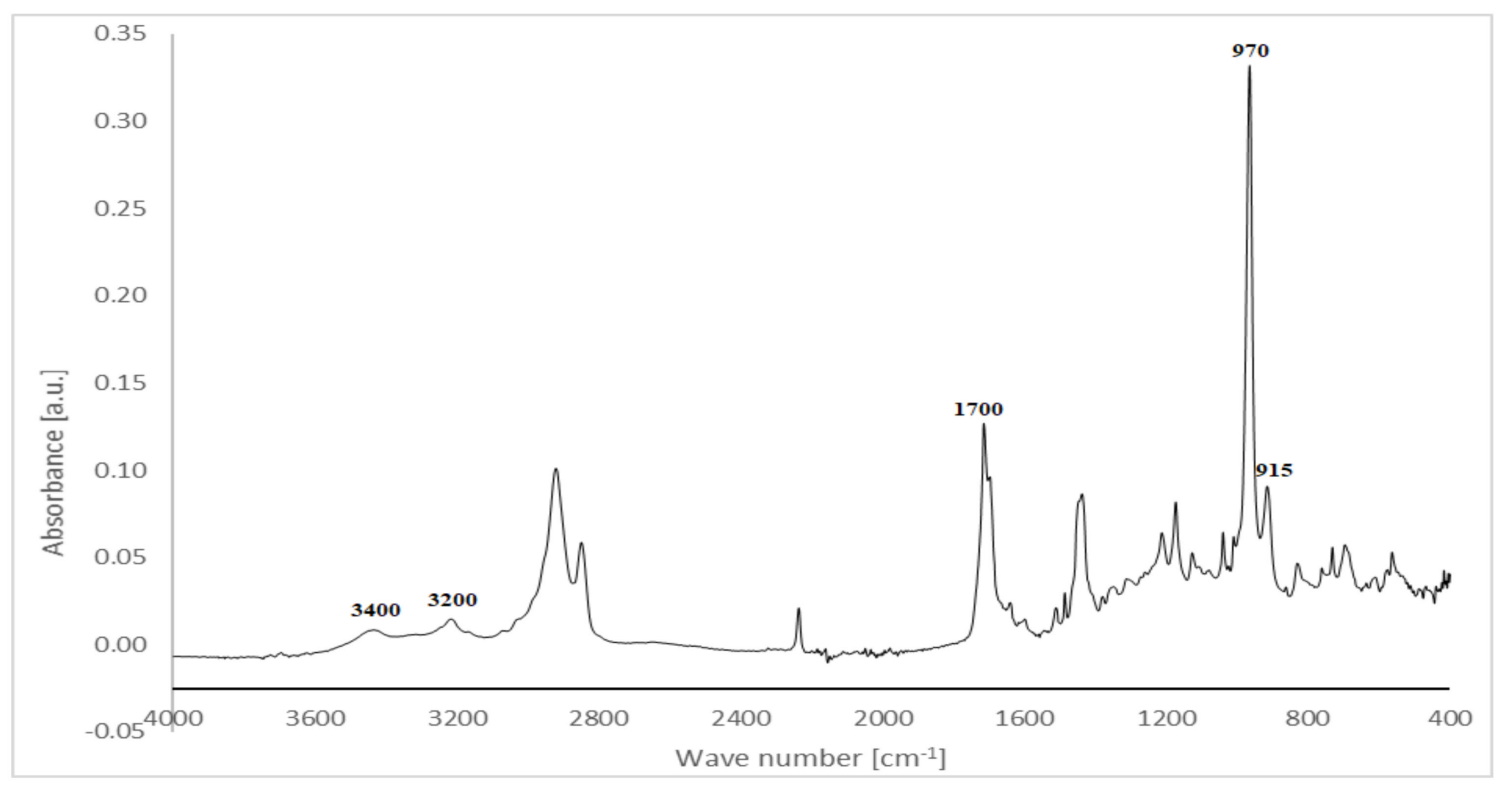
FTIR spectrum illustrating the way of crosslinking of XNBR with PO resin (XNBR_2PO).

Contrary to the crosslinking of SBR and NBR with the resin, in the FTIR spectrum of crosslinked XNBR (Figure 10) the absorption peak at 1740 cm^−1^, indicating the thermal decomposition product of the resin (6–8), does not disappear for rubber crosslinked with PO, but shifts to lower wavelength 1680–1700 cm^−1^, indicating their XNBR origin and probably representing the rubber crosslinking process. Characteristic absorption peaks, originating from hydroxyl groups at 3200–3400 cm^−1^, C-H bending vibrations in pendant double bonds at 970 cm^−1^ and 915 cm^−1^ arise in both cases, confirming the proposed mechanism of crosslinking XNBR with PO resin.

### 3.3. Mechanical Properties of Rubber

**Figure 11:**
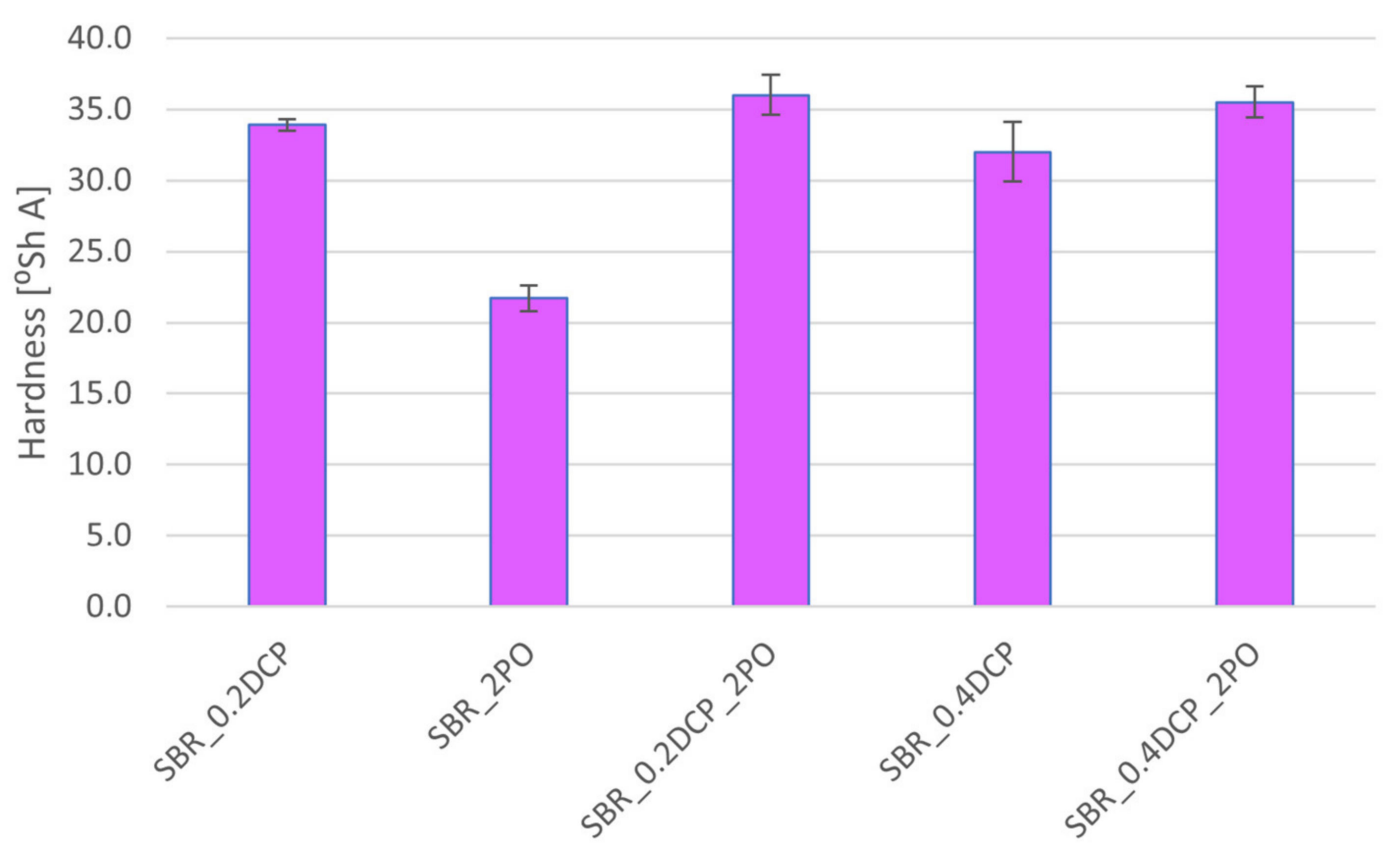
Influence of peroxide curing system modification on hardness of SBR samples.

**Figure 12:**
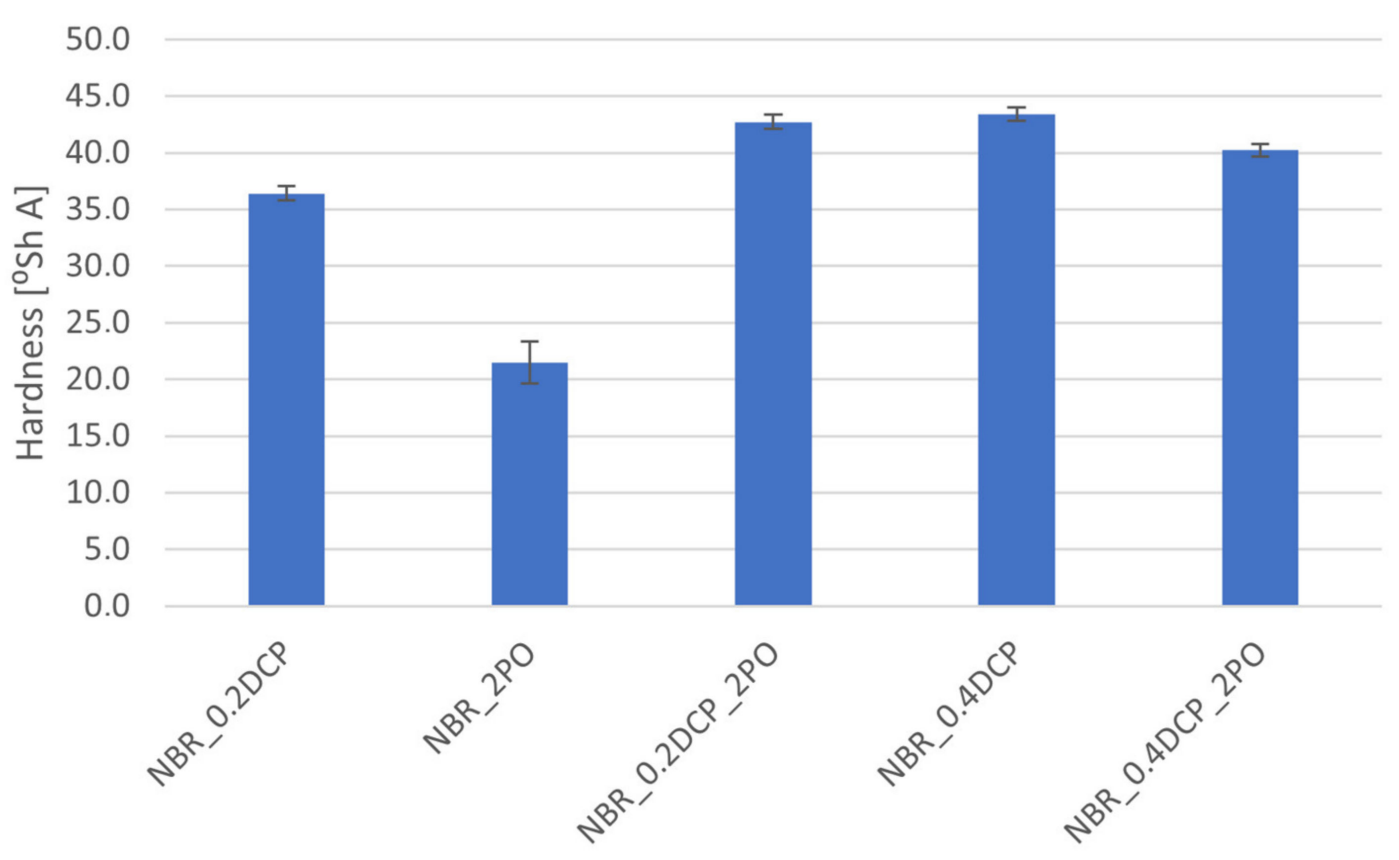
Influence of peroxide curing system modification on hardness of NBR samples.

**Figure 13:**
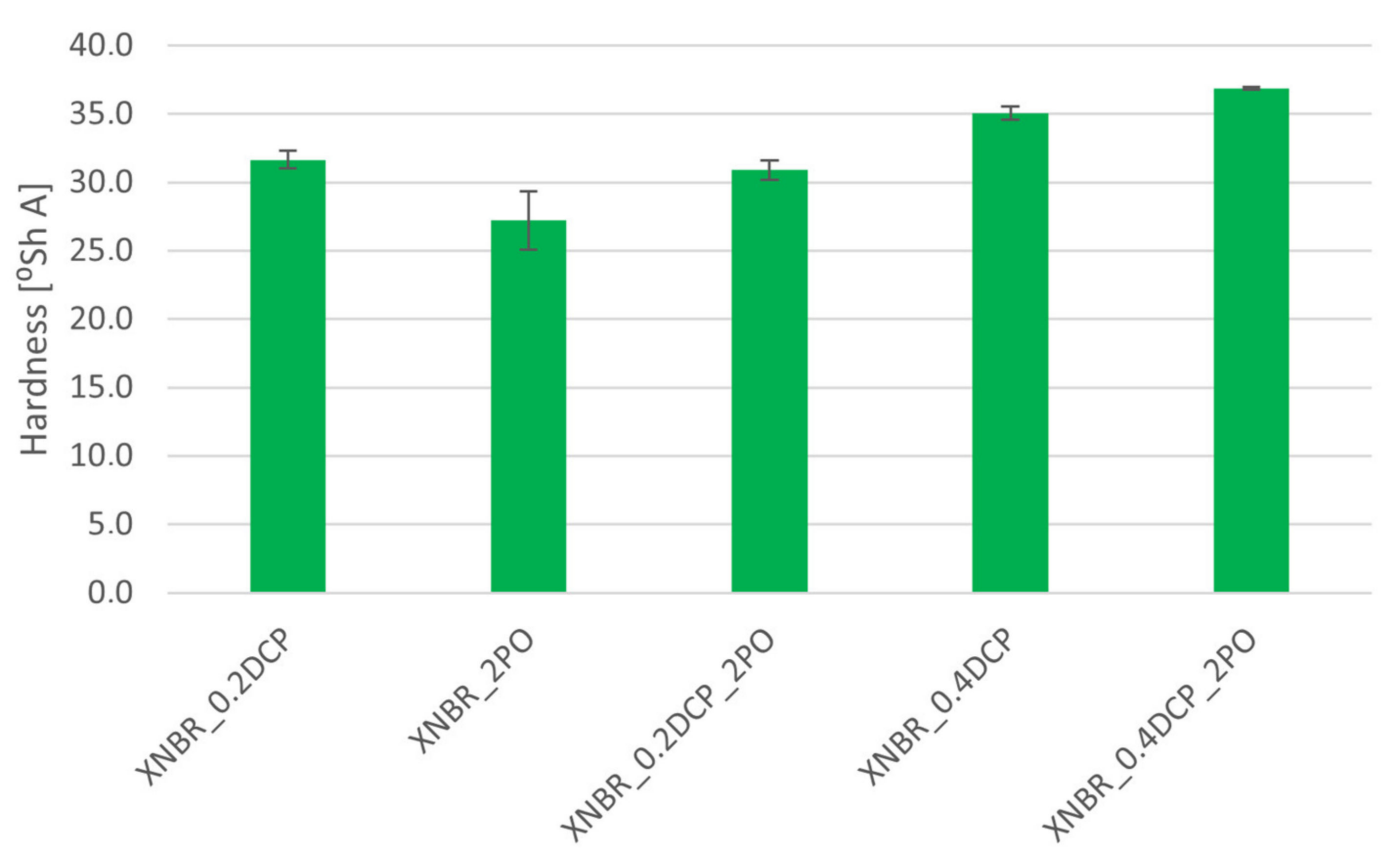
Influence of peroxide curing system modification on hardness of XNBR samples.

The mechanical properties of the crosslinked rubber samples are demonstrated in Table 3 and Table 4 and Figure 11, Figure 12 and Figure 13.

#### 3.3.1. Tensile Strength (TS)

The influence of the crosslinking system on the mechanical strength of the crosslinked samples tested depends on the type of rubber. For SBR, there is a general trend that systems containing both an organic peroxide and a modified resin exhibit higher tensile strength than those based solely on DCP. The difference is most apparent for samples crosslinked with 0.2 DCP and 0.2 PO. Elongation at the break values indicates that rubbers crosslinked with the mixed systems (DCP/resin) demonstrate a slightly higher strength but less flexibility than samples crosslinked only with DCP. Rubbers crosslinked with PO only exhibit indeed much lower strength but associated with higher elongation at break. Increasing DCP content results in an increase of the mixed system crosslinked samples stiffness, but their tensile strength does not improve, due to significant reduction in elongation at break.

In the NBR-based samples, there are no significant differences between stress at break for mixed systems and systems based only on DCP. However, the former have the highest TS value. Contrary to the case of samples based on SBR, the use of PO resin alone as a crosslinking agent reduces the mechanical strength of the rubber with an increase in its elongation at break. In general, however, the mechanical parameters of the NBR vulcanizates tested are inferior to the corresponding composition of SBR-based systems.

In the group of samples based on XNBR, those based on PO resin alone showed the highest flexibility. Using a system with conventional crosslinking agents in the amount of 0.2 phr together with 2 phr of PO made the samples’ elongation at break decrease and the accompanying stress increase.

#### 3.3.2. Tear Resistance (TES)

None of the samples crosslinked with a resin alone has been torn, similar to the XNBR compounds crosslinked with 0.2 phr of DCP and the resin. For all the rubbers tested, it is observed that the addition of PO resin generally reduces the force needed to tear the sample. The exception are rubbers containing 0.4 phr of DCP and 2 phr of PO as a crosslinking system, for which the tear force increases. Therefore, it can be concluded that the introduction of the modified resins into the DCP crosslinking system improves the tear resistance of the crosslinked rubbers tested.

#### 3.3.3. Hardness

The impact of the modification applied to the peroxide crosslinking system on the hardness of the rubber samples tested is presented in Figure 11, Figure 12 and Figure 13.

The addition of the modified resin to the rubbers tested, in combination with organic peroxide, causes a slight hardening of the samples, most evident in the case of those ones based on SBR. In turn, the use of PO resin alone reduces rubber hardness, especially SBR and NBR, indicating a plasticizing effect. In the systems based on XNBR, the observed changes to the rubber hardness as a result of the modification of the crosslinking system composition are the lowest among the rubber samples tested.

### 3.4. Adhesion

**Figure 14:**
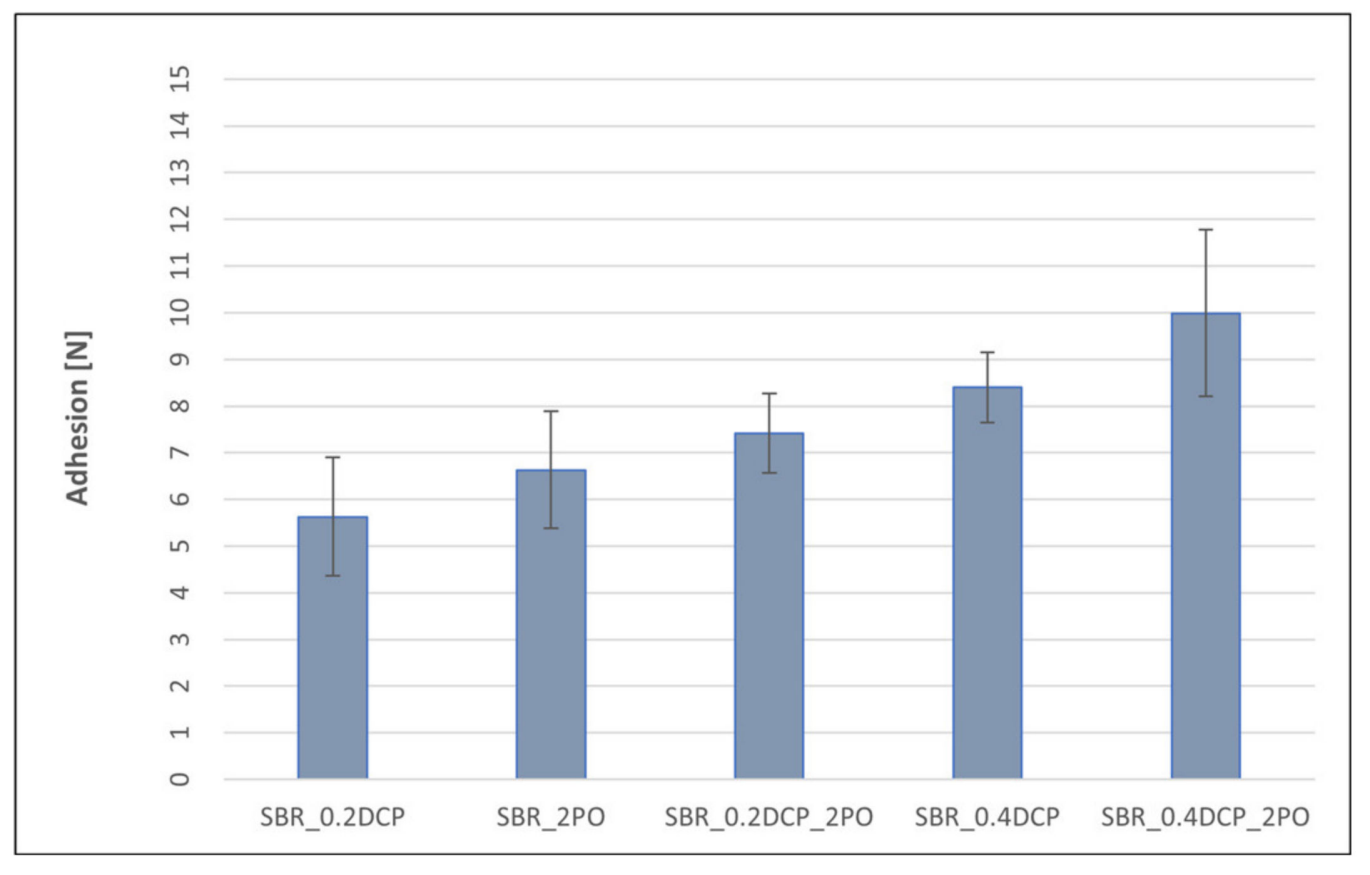
Influence of the modified resin addition on adhesion between peroxide SBR vulcanizates and silver wires.

**Figure 15:**
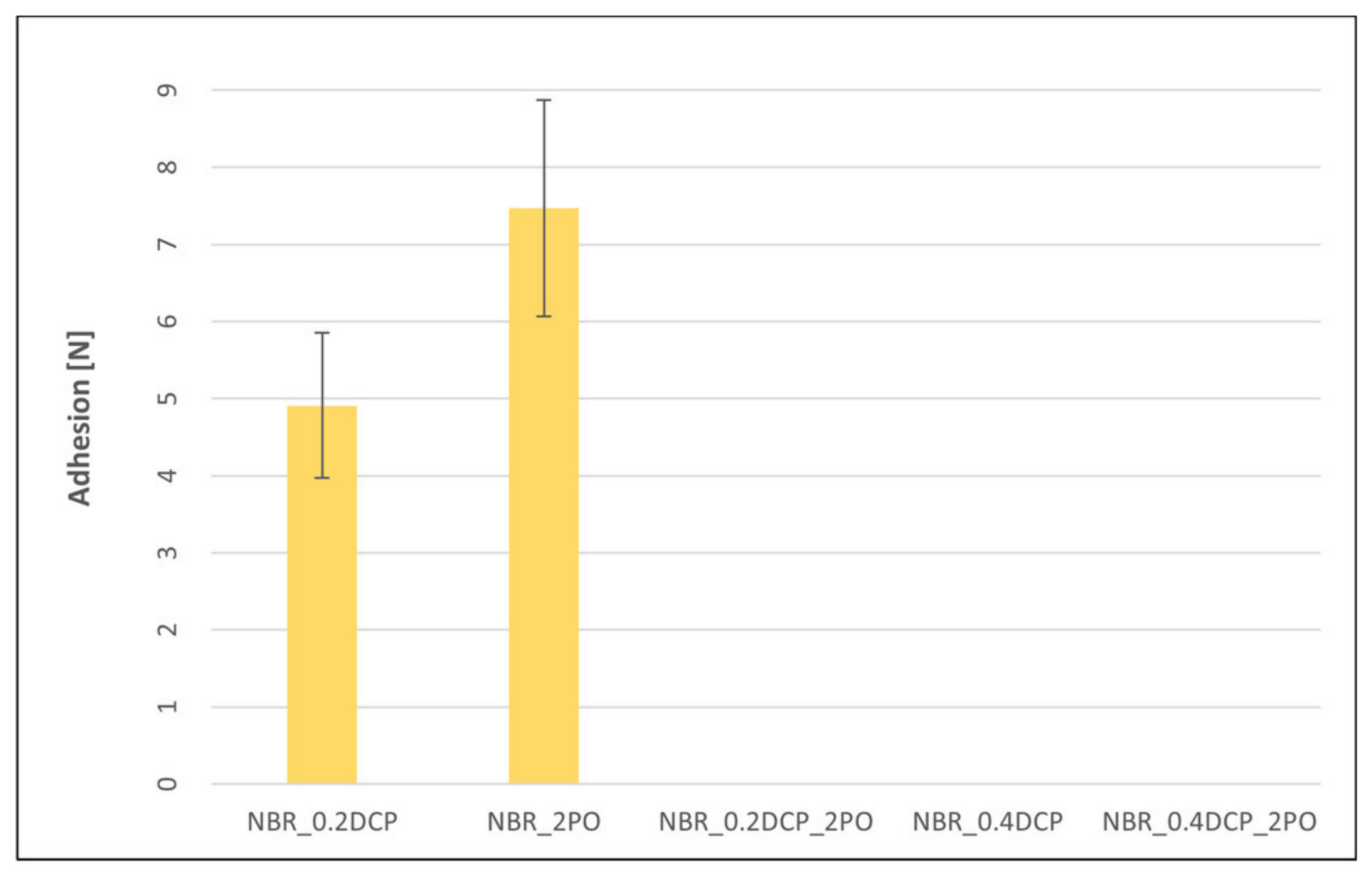
Influence of the modified resin addition on adhesion between peroxide NBR vulcanizates and silver wires (missing bars indicate on the lack of adhesion).

**Figure 16:**
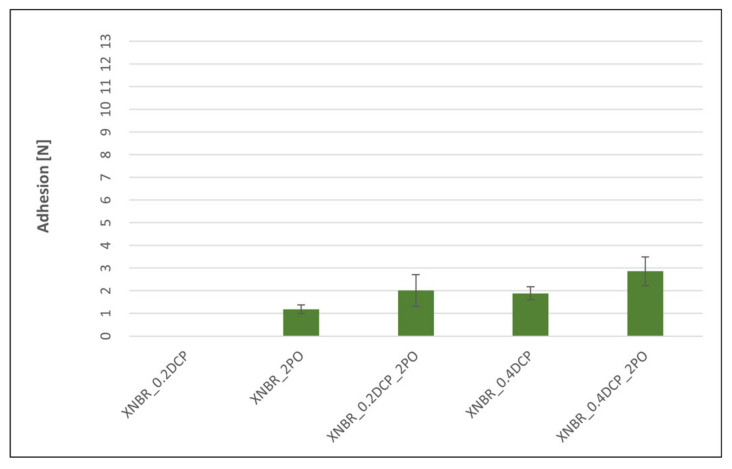
Influence of the modified resin addition on adhesion between peroxide XNBR vulcanizates and silver wires (missing bars indicate the lack of adhesion).

Due to the inability to measure the adhesion at the interface between the rubber matrix and the silver nanowires, a standard, the so-called “H” shape specimen method, often used for macroscopic wire/cord–rubber adhesion tests, was applied. Thus, information on the proadhesive potential of the resin additive in relation to selected synthetic rubbers crosslinked with dicumyl peroxide was expected. The results obtained are summarized in Figure 14, Figure 15 and Figure 16.

The addition of resin to peroxide rubber compounds increases adhesion between the SBR-based vulcanizates and the silver wires. In each case, taking into account the mechanical strength of rubbers (Table 3 and Table 4), a cohesive destruction of the connection should be expected. Even the use of PO resin alone (without peroxide) results in a significant increase in adhesion compared to relatively high adhesion for a sample crosslinked with 0.2 phr of DCP. For NBR vulcanizates no measurable adhesion between the rubber and the silver wires is observed, neither for mixed systems (peroxide/resin) nor for the sample crosslinked with 0.4 phr of DCP. On the other hand, the addition of PO resins alone, without peroxide, strongly improves NBR adhesion to the silver wires, just like for the SBR-based samples. The matter of the adhesion of silver wires to peroxide-cured XNBR looks similar to the case of SBR, except that the improvement obtained is much smaller.

## 4. Discussion

A synergistic effect of PO resin addition can be observed for the peroxide crosslinking of selected synthetic rubbers of technical importance: SBR, NBR and XNBR. It has been known since 1940s that phenol-formaldehyde resins (resoles) or halomethyl phenols in the presence of SnC_l4_⋯5H_20_ or FeC_l3_⋯6H_20_ and an acid catalyst can be used, even without peroxide’s presence, for the crosslinking of unsaturated rubbers such as NR, SBR, EPDM, IIR and NBR. However, it is worth mentioning that the scorch time of acrylonitrile-butadiene rubber is relatively short (low processing safety), subsequent to the very fast curing observed in this case. Crosslinking with resins may also be applicable to active hydrogen-containing rubbers such as Hypalon, acrylic rubber or XNBR [33]. Nevertheless, the crosslinking of rubbers with systems composed of peroxides and/or peroxy derivates of epoxy resins or the properties of their vulcanizates have seldom been studied. 

For mixed systems containing DCP and PO resin, it is often observed that the crosslink density of rubbers is higher than would result from the addition of DCP or resin alone. This affects the mechanical properties of the cured rubbers. The introduction of the resin into the peroxide curing system does not cause drastic changes to the mechanical strength of the rubbers studied; however, it can reduce their elongation at break. It was also observed that the addition of PO resin reduces the tear strength of the samples. The exceptions are the systems containing 0.4 phr of DCP and 2 phr of PO, for which TES increases. With a few exceptions, the addition of the modified resin also has no significant effect on the peroxide curing parameters of the rubbers tested. Moreover, the addition of the modified resin also results in a significant increase in adhesion between the SBR rubber and the silver wire, which can be used to help the research devoted to nanocomposites filled with silver nanowires, which can be used as mechanical stress sensors [34]. A slightly smaller improvement in adhesion has been observed for XNBR, whereas a lack of adhesion has been detected for NBR. Nevertheless, curing NBR with PO resin alone (without DCP) causes a significant increase in its adhesion to silver wires, but unfortunately the mechanical properties of the vulcanizates disqualify any application. 

Rather surprisingly there is not much information about silver–polymer interface in the subject literature. Some information can be find for fluorine polymers protecting silver mirrors, applied in concentrators for solar thermal systems [35,36]. Despite the great interest devoted to conductive epoxy adhesives filled with silver microplates or generally polymer–silver nanocomposites, the problem of silver–polymer interface has not been discussed. The authors mainly focused on the biocidal properties of the nanocomposites. Silver oxide, being a catalyst in the synthesis of epoxides [37], should not react with them. Apart from silver oxides, present on the surface of silver wires, hydroxyl groups are also potentially reactive towards epoxides. However, in the macroscale silver hydroxide is unstable due to the favorable energetics for the oxide formation. Nevertheless, some authors claim the presence of hydroxyl groups on the surface of oxidized silver [38], which could react with epoxides. Anyway, there are dipole–dipole interactions left that should be stronger in case of aromatic groups. Indeed, the highest adhesion between silver rods and rubber was obtained for SBR, which is less polar than NBR or XNBR. In most of the investigated systems, adding PO epoxide containing resin increases adhesion to silver, which can be attributed to the appearance of the adhesive component associated with the formation of the chemical bonds involving -OH groups [39], present in its structure. In the case of the phenylic groups present in SBR, another important chemical process along metal–polymer interfaces is worth considering, namely the interaction of the 4d electrons from transition metals such as silver with aromatic rings (π-electron) in polymers resulting in the formation of π-4d electrons’ interaction [40]. Indeed, the highest adhesion between silver rods and rubber was obtained for SBR, containing aryl group, absent in case of NBR or XNBR. The lack of measurable adhesion between the silver rod and the stronger crosslinked NBR is most likely a physical effect, associated mainly with different thermal shrinkage of the metal and the rubber after vulcanization. The unexpected, generally low adhesion of XNBR to silver will be the topic of future investigations.

To sum up, among the rubbers studied, only SBR crosslinked with DCP and the addition of PO can be recommended as a potential matrix for nanocomposites filled with silver nanowires, serving e.g., as a potential sensor matrix for stress testing.

## 5. Conclusions

The above results clearly demonstrate the potential of using a monoperoxy derivative of epoxy resin (PO) as a multifunctional additive to synthetic rubbers in order to improve their adhesion to metal, promoting cross-linking and modifying crosslinks’ structures. Despite general lack of information about silver–polymer interface in the subject literature, the application of PO, together with dicumy peroxide (DCP) allows for the modification of a polymer matrix to be “tailored” for silver. The modification was shown to be especially effective for styrene-butadiene rubber (SBR), despite SBR having the lowest polarity among the rubbers studied. The research performed concerned silver; nevertheless, there are many indications that the modification could also work for other metals. Recent studies [41] have shown theoretical models that can help to quickly assess the effects of hyperelastic properties on the metal–rubber bond’s behavior and strength.

## Figures and Tables

**Table 1:** Composition of the rubber compounds studied (phr).

Mixture	Rubber	DCP	PO
SBR_0.2DCP	100	0.2	0
SBR_2PO	100	0	2
SBR_0.2DCP_2PO	100	0.2	2
SBR_0.4DCP	100	0.4	0
SBR_0.4DCP_2PO	100	0.4	2
NBR_0.2DCP	100	0.2	0
NBR_2PO	100	0	2
NBR_0.2DCP_2PO	100	0.2	2
NBR_0.4DCP	100	0.4	0
NBR_0.4DCP_2PO	100	0.4	2
XNBR_0.2DCP	100	0.2	0
XNBR_2PO	100	0	2
XNBR_0.2DCP_2PO	100	0.2	2
XNBR_0.4DCP	100	0.4	0
XNBR_0.4DCP_2PO	100	0.4	2

2 phr of monoperoxy derivative of epoxy resin (PO) with its peroxide content is equivalent to 0.2 phr of DCP (dicumyl peroxide—98%, Merck (Germany)), based on the weight content of peroxide groups present in the both chemical reagents. SBR—styrene-butadiene rubber: styrene content 23.5%, Ker 1502, Synthos (Poland); NBR—acrylonitrile-butadiene rubber: acrylonitrile content 17%, Perbunan 1846, Arlanxeo (Germany); XNBR—carboxylated acrylonitrile-butadiene rubber: acrylonitrile content 27%, carboxylic acid content 7%, Krynac X 750, Arlanxeo (Germany).

**Table 2:** Curing parameters of the rubber compounds studied.

Rubber Mix	t_90_ [min]	MH [dNm]	ML [dNM]	ΔM [dNm]
SBR_0.2DCP	17	4.8	1.0	3.8
SBR_2PO	5	2.4	1.0	1.4
SBR_0.2DCP_2PO	17	5.7	1.0	4.7
SBR_0.4DCP	17	7.6	1.0	6.6
SBR_0.4DCP_2PO	17	8.1	1.0	7.1
NBR_0.2DCP	25	5.9	1.4	4.5
NBR_2PO	30	2.4	1.3	1.1
NBR_0.2DCP_2PO	23	8.2	1.2	7.0
NBR_0.4DCP	21	9.0	1.3	7.7
NBR_0.4DCP_2PO	22	7.7	1.4	6.3
XNBR_0.2DCP	25	2.2	0.5	1.7
XNBR_2PO	33	1.3	0.5	0.8
XNBR_0.2DCP_2PO	23	2.7	0.5	2.2
XNBR_0.4DCP	21	4.9	0.5	4.4
XNBR_0.4DCP_2PO	20	5.0	0.5	4.5

t_90_—vulcanization time, MH and ML—max. and min. torque adequately, ΔM = MH − ML—torque increase.

**Table 3:** Mechanical properties of the rubber samples tested under elongation.

Sample	M_100_ [MPa]	M_200_ [MPa]	M_300_ [MPa]	Tensile Strength [MPa]	Elongation at Break [%]
SBR_0.2DCP	0.57 ± 0.11	0.96 ± 0.08	1.16 ± 0.06	1.59 ± 0.21	510 ± 89
SBR_2PO	0.36 ± 0.06	0.48 ± 0.06	0.58 ± 0.06	1.74 ± 0.13	979 ± 47
SBR_0.2DCP_2PO	0.62 ± 0.05	0.99 ± 0.08	1.21 ± 0.08	1.78 ± 0.28	313 ± 92
SBR_0.4DCP	0.72 ± 0.08	1.16 ± 0.00	1.43 ± 0.08	1.51 ± 0.16	313 ± 14
SBR_0.4DCP_2PO	0.73 ± 0.09	1.20 ± 0.00	-	1.50 ± 0.24	222 ± 28
NBR_0.2DCP	0.81 ± 0.07	1.26 ± 0.15	1.55 ± 0.11	1.68 ± 0.12	287 ± 49
NBR_2PO	0.45 ± 0.03	0.62 ± 0.06	0.79 ± 0.09	1.16 ± 0.06	562 ± 101
NBR_0.2DCP_2PO	0.85 ± 0.02	1.24 ± 0,06	1.63 ± 0.12	1.65 ± 0.09	298 ± 21
NBR_0.4DCP	1.04 ± 0.14	-	-	1.49 ± 0.26	169 ± 20
NBR_0.4DCP_2PO	0.94 ± 0.11	1.27 ± 0.13	-	1.47 ± 0.19	203 ± 68
XNBR_0.2DCP	0.67 ± 0.03	0.77 ± 0.03	0.83 ± 0.03	1,86 ± 0,17	1070 ± 76
XNBR_2PO	0.60 ± 0,01	0.64 ± 0.02	0.65 ± 0.03	>1.33 ± 0.05	*
XNBR_0.2DCP_2PO	0.65 ± 0.02	0.75 ± 0.04	0.82 ± 0.07	2.41 ± 0.26	956 ± 41
XNBR_0.4DCP	0.76 ± 0.03	0.92 ± 0.05	1.06 ± 0.08	2.51 ± 0.29	653 ± 82
XNBR_0.4DCP_2PO	0.71 ± 0.00	0.88 ± 0.02	1.01 ± 0.07	1.68 ± 0.66	530 ± 101

M_100_, M_200_ and M_300_—mechanical moduli of the materials respectively at 100, 200 and 300% of elongation. * Samples not broken under max. Experimental range of extension (450 mm).

**Table 4:** Tear strength of the rubber samples tested.

Sample	Fmax. [N/mm]	Fmin. [N/mm]
SBR_0.2DCP	6.05	5.78
SBR_2PO	no tearing	
SBR_0.2DCP_2PO	4.88	3.96
SBR_0.4DCP	3.5	2.3
SBR_0.4DCP_2PO	3.0	1.0
NBR_0.2DCP	5.96	3.18
NBR_2PO	no tearing	
NBR_0.2DCP_2PO	2.83	1.66
NBR_0.4DCP	2.66	1.72
NBR_0.4DCP_2PO	3.36	2.73
XNBR_0.2DCP	no tearing	
XNBR_2PO	no tearing	
XNBR_0.2DCP_2PO	no tearing	
XNBR_0.4DCP	7.71	5.75
XNBR_0.4DCP_2PO	8.22	5.97

## Data Availability

The raw/processed data required to reproduce these findings cannot be shared at this time due to technical or time limitations.
